# Redox balance, metabolic fingerprint and physiological characterization in contrasting North East Indian rice for Aluminum stress tolerance

**DOI:** 10.1038/s41598-019-45158-3

**Published:** 2019-06-18

**Authors:** Jay Prakash Awasthi, Bedabrata Saha, Jogeswar Panigrahi, Emiko Yanase, Hiroyuki Koyama, Sanjib Kumar Panda

**Affiliations:** 1 Assam University, Department of Life Science and Bioinformatics, Plant Molecular Biotechnology Lab, 788011 Silchar, India; 2 Khallikote University, Department of Bioscience and Bioinformatics, 760001 Berhampur, India; 3 Gifu University, Faculty of Applied Biological Sciences, 5011193 Gifu, Japan

**Keywords:** Mass spectrometry, Abiotic

## Abstract

Aluminum (Al) toxicity is a serious problem for rice crop productivity in acidic soils worldwide. The present work was conducted to look out for the alteration in ROS homeostasis; metabolic fingerprint; and morphology in two contrasting *Indica* rice cultivars of North East India (NE India) to Al toxicity. Al stress led to excess accumulation of ROS (H_2_O_2_ and O_2_^−^), and this in turn induced ROS mediated cellular damage, as indicated by lipid peroxidation both qualitatively as well as quantitatively. This excessive ROS production also led to significant reduction in chlorophyll content and stomatal conductance. This was followed by the loss of photosynthetic efficiency as detected by chlorophyll fluorescence. This excessive damage due to ROS prompted us to check the anti-oxidative machinery. Antioxidants, especially enzymes (SOD, APX, POX, GR, CAT, DHAR, MDHAR) are very important players in maintenance of ROS homeostasis. In tolerant variety Disang, higher activity of these enzymes and vice versa in sensitive variety, was observed in response to Al treatment. The non-enzymatic antioxidants (proline, ascorbate and glutathione) also showed similar trend. Though the tolerant variety showed strong anti-oxidative machinery, it was unable to completely nullify the stress experienced by the seedlings. Organic acids are also important players in detoxification of Al stress through efflux in the rhizosphere. In tolerant genotype, citrate exudate was found to be more when compared to sensitive genotypes on exposure to high dose of Al. This is supported by higher abundance of FRDL4, a citrate transporter. Not only FRDL4, other stakeholders for Al stress response like ART1 and ALS1 depicted prominent transcript abundance in the tolerant variety. In conclusion, through this study detailed physiological and metabolic characterisation of two contrasting *Indica* rice varieties Disang and Joymati, native to NE India for Al tolerance was performed for the very first time.

## Introduction

Aluminum (Al) is the third most abundant, and ubiquitously distributed metallic element in the earth crust^1^. The toxicity of Al depends on soil acidity. Acid soil comprises of approximately 30% of land area worldwide and 50% of world’s arable land has pH below 5. Rice (*Oryza sativa* L.) has been found out to be comparatively the most Al-tolerant, among cereal crops^2^. Though more tolerant, Al toxicity has its effect felt on rice too. Rice constitutes the food for half of the population worldwide, making it the world’s most important cereal crop. About 90% of total rice production is cultivated and consumed in Asia. Al phyto-toxicity causes speedy suppression of root elongation, and thus root relative elongation rate has functioned as a distinctive marker for degree of toxicity and tolerance in plants^3^. Al toxicity due to acidic soil, induces reactive oxygen species (ROS), which in turn triggers lipid peroxidation, limits ion transport capacity, affects membrane fluidity and causes protein oxidation etc. in plants^4–6^.

Determination of redox status is essential to predict the metabolic activities and the health of cells. Pyridine nucleotides such as NADH-NAD^+^ and NADPH-NADP^+^ are fundamental players in signalling through ROS molecules^7^. The balance between the oxidized and reduced forms, *ie*. NADH/ NAD^+^ ratio is an indicant of the redox status. ROS triggers deleterious reactions and plants have thus developed a complex antioxidant system to preclude the catastrophic consequences of ROS^5^. Excess ROS molecules oxidizes cellular macromolecules finally directing to cell death. H_2_O_2_ is a versatile molecule of the ROS network, generated in part by superoxide dismutase (SOD) from superoxide anion under stressful conditions^8^. One of the key role of H_2_O_2_ in Al stress signalling might be the possible involvement in lignin synthesis through peroxidase activity. H_2_O_2_ has got crucial function as an electron donor for coniferyl alcohol peroxidase, taking part in lignin formation. Lignin deposition leads to decreased cell elongation^9^. Hence plant cells possesses a defensive system composed of enzymatic and non-enzymatic antioxidants that help to detoxify the ROS.

Though ROS molecules are unavoidable by-products of oxygenic photosynthesis, excess accumulation of the former leads to damage in photosynthetic apparatus^10^. Thus chlorophyll fluorescence is a powerful tool to obtain a full picture of plants to environmental cues^11^.

In order to isolate the affected cells from the healthy one’s plants resort to callose deposition in plasmodesmata. Callose is a β-1, 3-glucan, that is very rarely detected within plant cells and only known to be needed in a few very specific developmental procedures. Al-induced responses, leads to speedy accumulation of callose, which can be detected in plants within a few hours of exposure to Al. As its accumulation is so closely connected to Al stress, callose deposition has been a useful determinant for evaluating manifestations due to Al toxicity^11^.

Al-responsive genes play crucial role in fine tuning of stress response, be it nucleus localized transcription factors or functional genes whose products directly mediate in Al tolerance. ART1 (Aluminum Resistance Transcription Factor 1) caters to a vital role in Al stress response in rice. ART1 regulates genes that are crucial in eliciting response to Al toxicity^12^. Whereas, ALS1 (Aluminum Sensitive 1), a half-size ABC transporter, is involved in the vacuolar sequestration of Al and is localized in the tonoplast of root cells^13^. Since organic acids have a crucial role in Al detoxification, the genes encoding organic acid transporters are of utmost importance. Three citrate transporters has been reported in rice; *Os*FRDL1, *Os*FRDL2, *Os*FRDL4 which belongs to MATE (Multidrug And Toxic compound Extrusion) group. Out of the three, only *Os*FRDL4 has been accounted to be responsible for Al stress mediated citrate extrusion^14^.

Once being sensitized to Al toxicity, plants in its defence response secrete organic acids both in cytoplasm and rhizosphere. In the cytosol, these organic acids can form stable complexes with Al, thereby precluding the adherance of Al to cellular components. Whereas, organic acids in the rhizosphere also forms complexes with Al and prevents excess uptake of Al ions by the root apex cells. Hence, Al-stimulated synthesis of organic acid, has been widely examined in multiple studies^15^. This work was carried out to provide a differential insight into redox, metabolic and physiological fingerprint of two contrasting rice varieties of North East India to Al stress in acidic condition.

## Results

### Plant growth under Al stress

**Figure 1:**
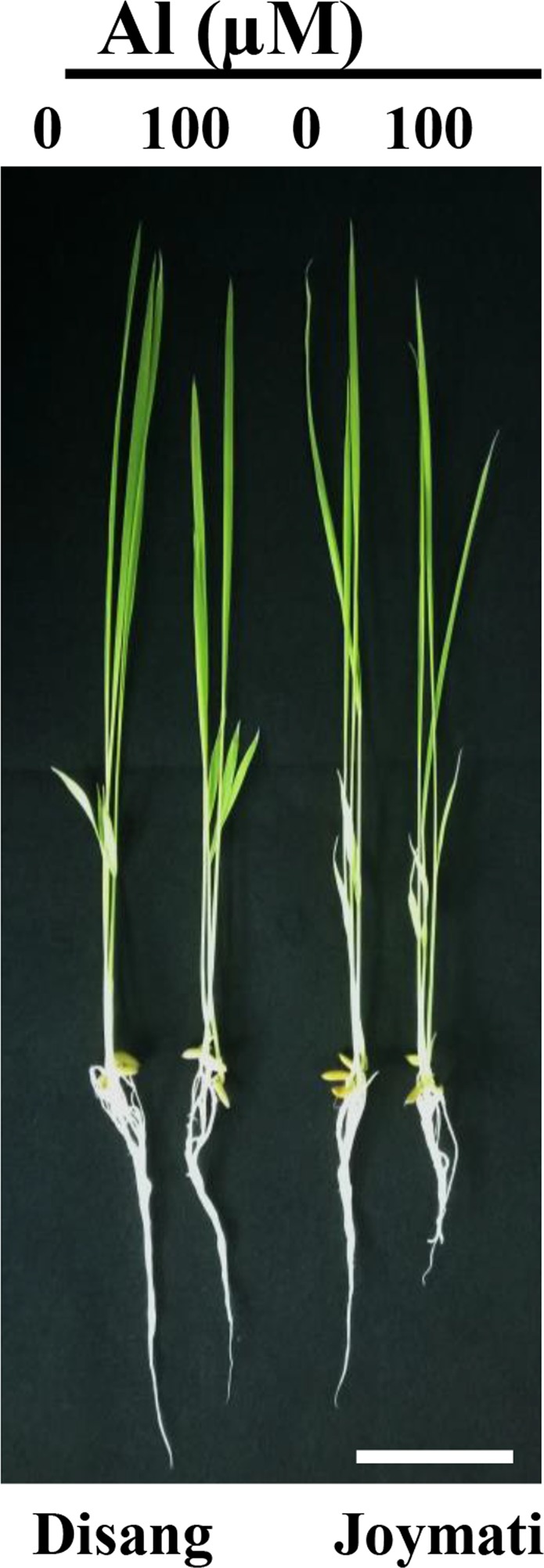
Effect of Al on root growth of rice seedlings (Al tolerant; Al sensitive) the seedling were grown for 6 day in nutrient solution After that treatment in solution containing 0, 100 µM AlCl_3_ in 0.5 mM CaCl_2_ at pH 4.5 for 48 h was exerted. (Bar represent the 1 cm).

Al stress dose on rice seedlings led to morphological changes and growth inhibition (Figs 1, S1). In stressed plants, root length reduced significantly, irrespective of cultivar. The cultivar Disang was the quickest growing with most eminent root biomass, whereas Joymati showed much reduction after 48 h (Fig. 1). On the contrary, shoot biomass production didn’t show significant reduction due to stress. After five days, treated plants showed more damage in shoot of Joymati variety when compared to Disang.

### Oxidative stress in rice on Al^3+^ toxicity

#### ROS accumulation

**Figure 2:**
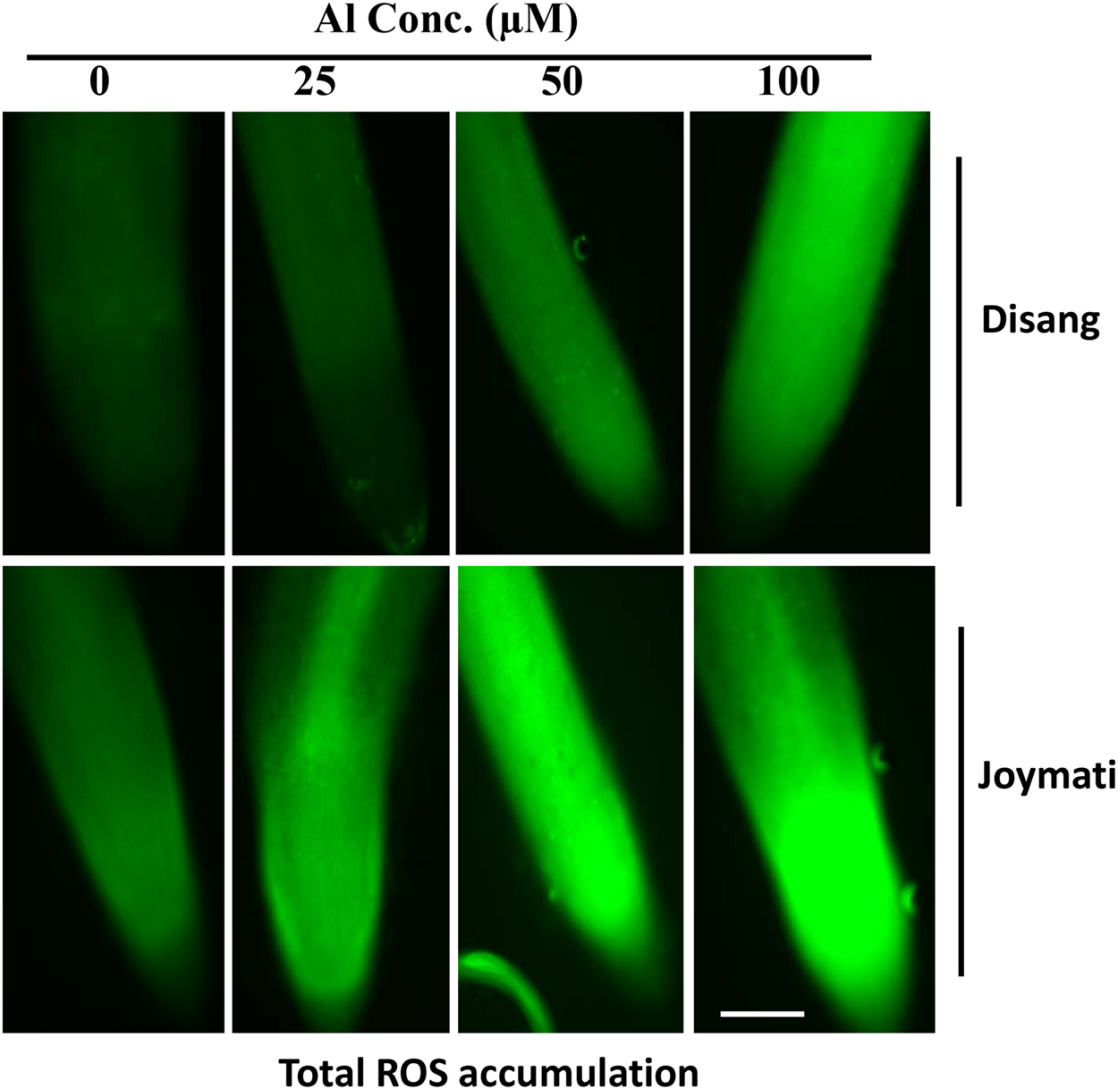
Microscopic observation of total ROS accumulation in the root tip region was visualized by 2′,7′-dichloro dihydrofluorescein diacetate (H_2_DCFDA) then observed under a fluorescence microscope. White bar indicates 100 μ. At least three biological replicates were performed. Representative images showing endogenous ROS production. Rice root were exposed to various Al treatments (0, 25, 50 and 100 µM) for 48 h.

Higher accumulation of ROS was observed under stress condition as revealed by staining with H_2_DCFDA dye. As indicated by staining intensity, accumulation was more in Joymati in response to Al treatment and low pH when equated to Disang (Fig. 2).

#### Determination of superoxide radical and hydrogen peroxide levels

**Figure 3:**
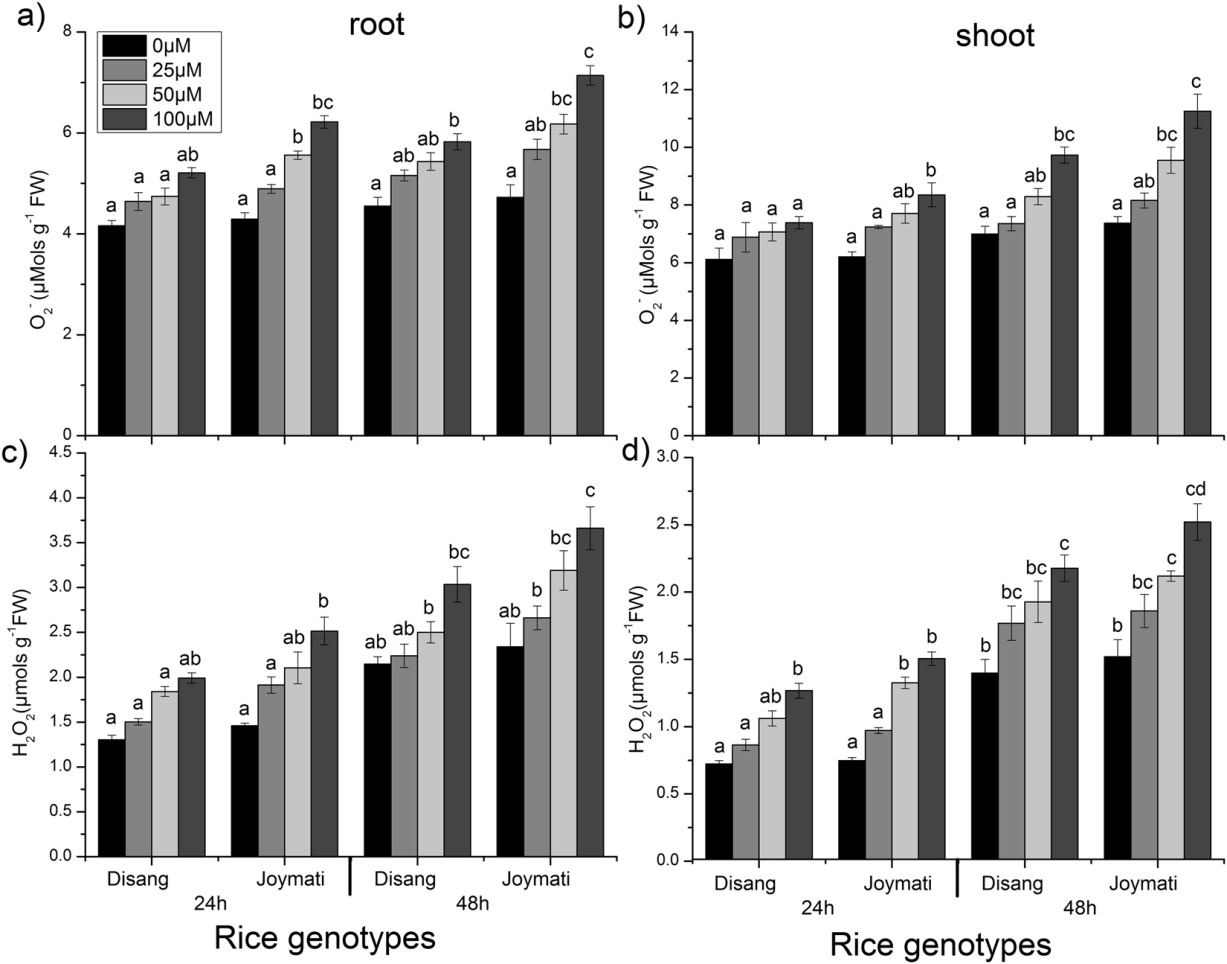
Effects of Al, on superoxide anion (O^2−^) and hydrogen peroxide (H_2_O_2_) content in the roots (**a**,**c**) and in the shoots (**b**,**d**) of the two rice varieties. Seedlings were exposed to 0, 25, 50,100 µM AlCl_3_, containing 0.5 mM CaCl_2_ (pH 4.5) for 48 h. Values are mean ± SE (n = 3) of three separate experiments. Means denoted by the same letter were not significantly different at P < 0.05 according to Duncan’s multiple range test.

Dose and time dependent superoxide (O_2_^−^) and hydrogen peroxide (H_2_O_2_) content under Al stress in both root (Fig. 3a) and shoot (Fig. 3b); was observed. The content of both ROS molecules was more in 100 µM of Al^3+^ in both root and shoot at 24 h and 48 h interval. Superoxide radical and hydrogen peroxide significantly increased at higher dose of Al stress. Joymati accumulated more of these free radicals when compared to Disang (Fig. 3).

### Measurement of damage due to oxidative stress in rice on Al^3+^ excess

#### Stomatal conductance and photosynthetic pigments of Al stressed seedlings

**Figure 4:**
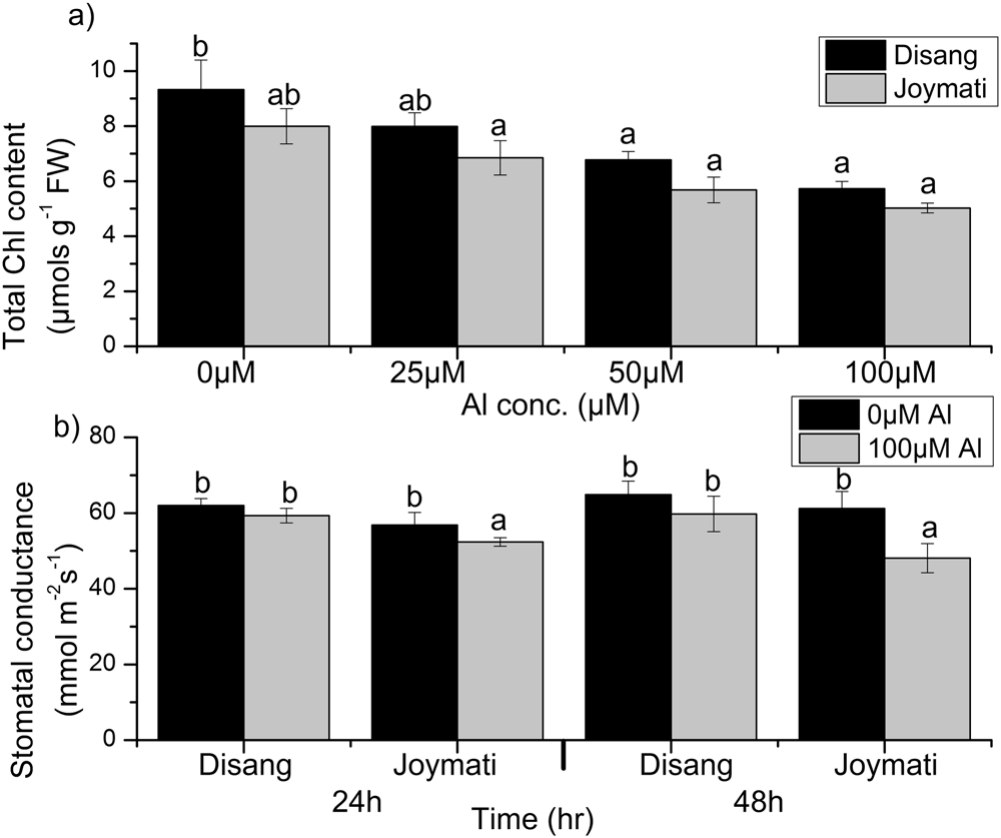
Effects of Al, on the total chlorophyll content (**a**) and stomatal conductance (**b**) in the shoot of the two rice varieties. Seedlings were exposed to AlCl_3_, containing 0.5 mM CaCl_2_ (pH 4.5) for 48 h. Values are mean ± SE (n = 3) of three separate experiments. Means denoted by the same letter were not significantly different at P < 0.05 according to Duncan’s multiple range test.

The photosynthetic pigment content such as total chlorophyll (Fig. 4a) chlorophyll a, chlorophyll b, and carotenoid content (Fig. S2) on Al stress was determined. Stomatal conductance decrease at higher concentration of Al, in Joymati was more when compared to Disang (Fig. 4b) after 48 h.

#### Chlorophyll fluorescence studies

The study of chlorophyll fluorescence parameters such as Fo, Fm, Fv/Fm, Fo, Fm′, Fv′/Fm′, Y(II), qP, qN, qL, NPQ, Y(NO), Y(NPQ) and ETR was conducted. The chlorophyll degradation in both cultivars was progressively correlated with maximum quantum yield of PSII (Fv/Fm) as well as the optimum quantum yield of PSII (Y(II)), leading to growth reduction. The correlation among the chlorophyll fluorescence parameters are presented in Table S1. The Fm, Fv/Fm, Y(II), Fm′, ETR, qP, qL, Y(NO), Fv′/Fm′ showed negative correlations while Y(NPQ), NPQ, Fo′, qN, Fo showed positively co- related in both cultivars to Al stress treatment (Table S1).

#### Measurement of protein oxidation and lipoxygenase on Al stress

Protein carbonylation was assessed in both root and shoot under Al stress condition. The result revealed that the stressed plants had higher carbonylated proteins. The level of carbonyl bound protein content enhanced after 24 h and 48 h interval in both root and shoot (Fig. S3). Lipoxygenase (LOX) enzyme activity evaluated in root and shoot, was elevated in higher concentration of Al for both cultivars (Fig. S3). In shoot, LOX activity increased significantly in higher dose at 24 and 48 h interval.

#### Lipid peroxidation

**Figure 5:**
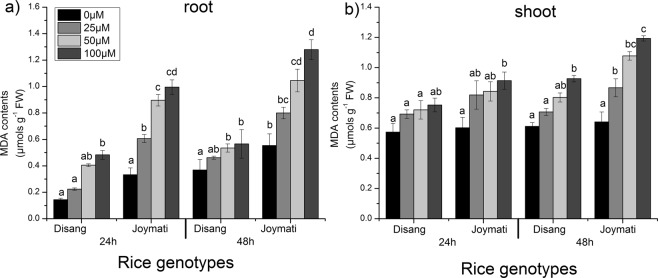
Effects of Al on the MDA content in root (**a**) and shoot (**b**) of the two rice varieties. Seedlings were exposed to 0.5 mM CaCl_2_ (pH 4.5) containing 0, 25, 50,100 µM AlCl_3_ for 48 h. Values are mean ± SE (n = 3) of three separate experiments. Means denoted by the same letter were not significantly different at P < 0.05 according to Duncan’s multiple range test.

MDA is typically used as a marker of levels of lipid peroxidation. Enhanced MDA content was observed in both varieties 24 and 48 h after Al treatment when equated with control irrespective of tissue samples. Joymati showed more lipid peroxidation in comparison to Disang in both root and shoot sample (Fig. 5). MDA is the ultimate produce of membrane lipid peroxidation and impacts membrane fluidity, induce protein degradation and confines the capability of ion transport, which at last leads to cell death.

#### Callose and lignin accumulation in Al stressed seedlings

**Figure 6:**
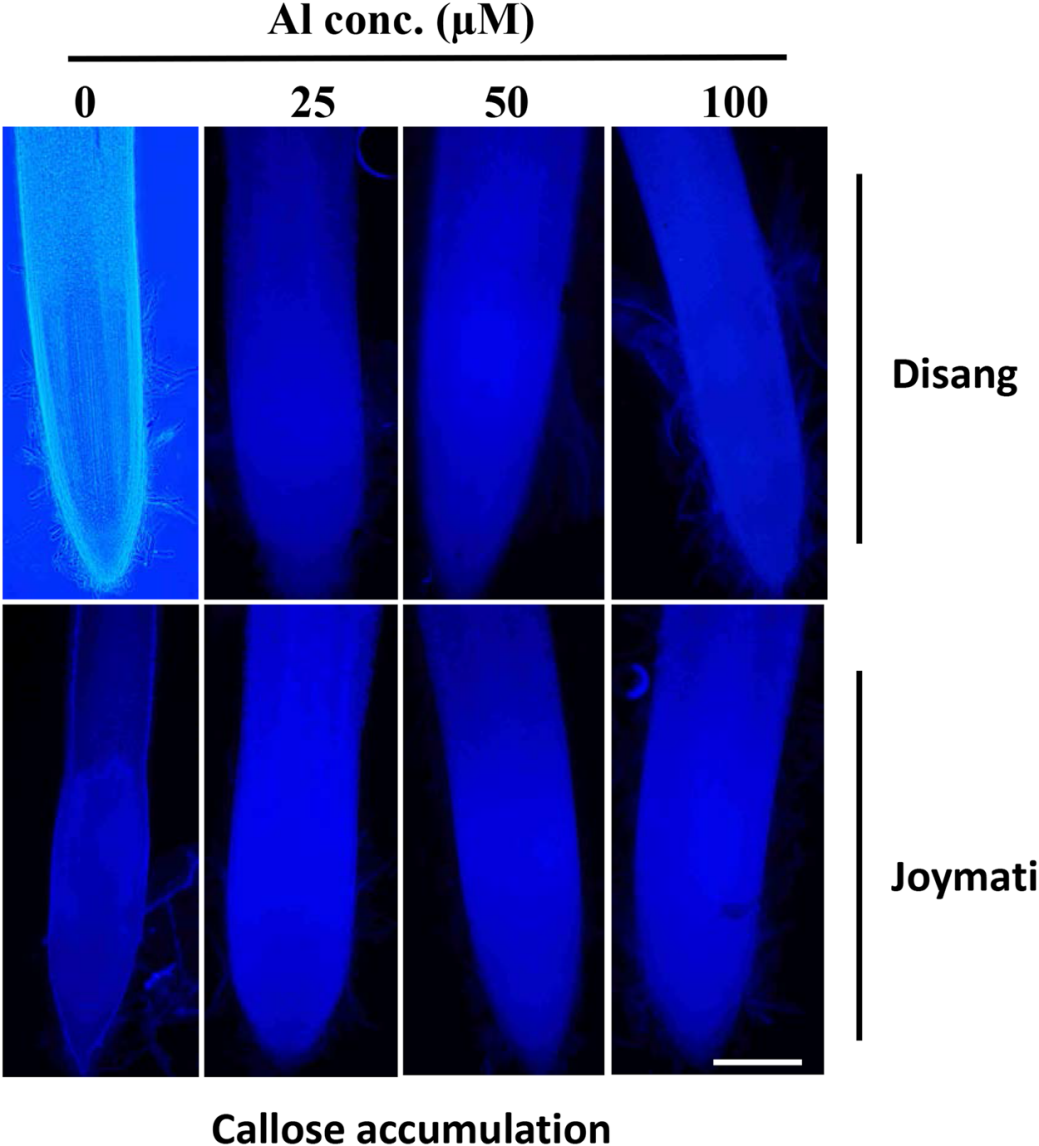
Microscopic observation of root tips exposed to Al stress. Callose accumulation in the root tip region was visualized by aniline blue then observed under a fluorescence microscope. White bar indicates 100 μ. At least three biological replicates were performed. Rice root were exposed to various Al treatments (0, 25, 50 and 100 µM) for 48 h.

Callose accumulation was determined in the rice root both qualitatively (Fig. 6) and quantitatively (Fig. S4). Significant changes in the accumulation was observed in sensitive cultivars under higher concentration of the Al stress when equated to Disang. There was a significant enhancement in lignin content under stress condition for Joymati in comparison to Disang at both intervals (Fig. S4).

#### Pot stress assay

The tolerance capability of both cultivars in soil under Al stress was ascertained. Though both varieties were significantly affected Joymati showed better tolerance phenotype to stress when compared to Disang (Fig. S5).

### Al^3+^ stress response in rice seedlings

#### Estimation of soluble protein and SDS PAGE profiling

Total protein was quantified to check the effects of Al in root and shoot of rice sample. In response to diverse dose of Al the total protein content was found altered. Root showed increased total protein content with the increase in Al dose (Fig. S6) whereas shoot showed decrease in total protein content (Fig. S7). SDS PAGE profiling of stressed and unstressed plant protein showed increase in band intensity of some bands (Fig. S7).

#### Determination of Glutathione, Ascorbate and Proline content

**Figure 7:**
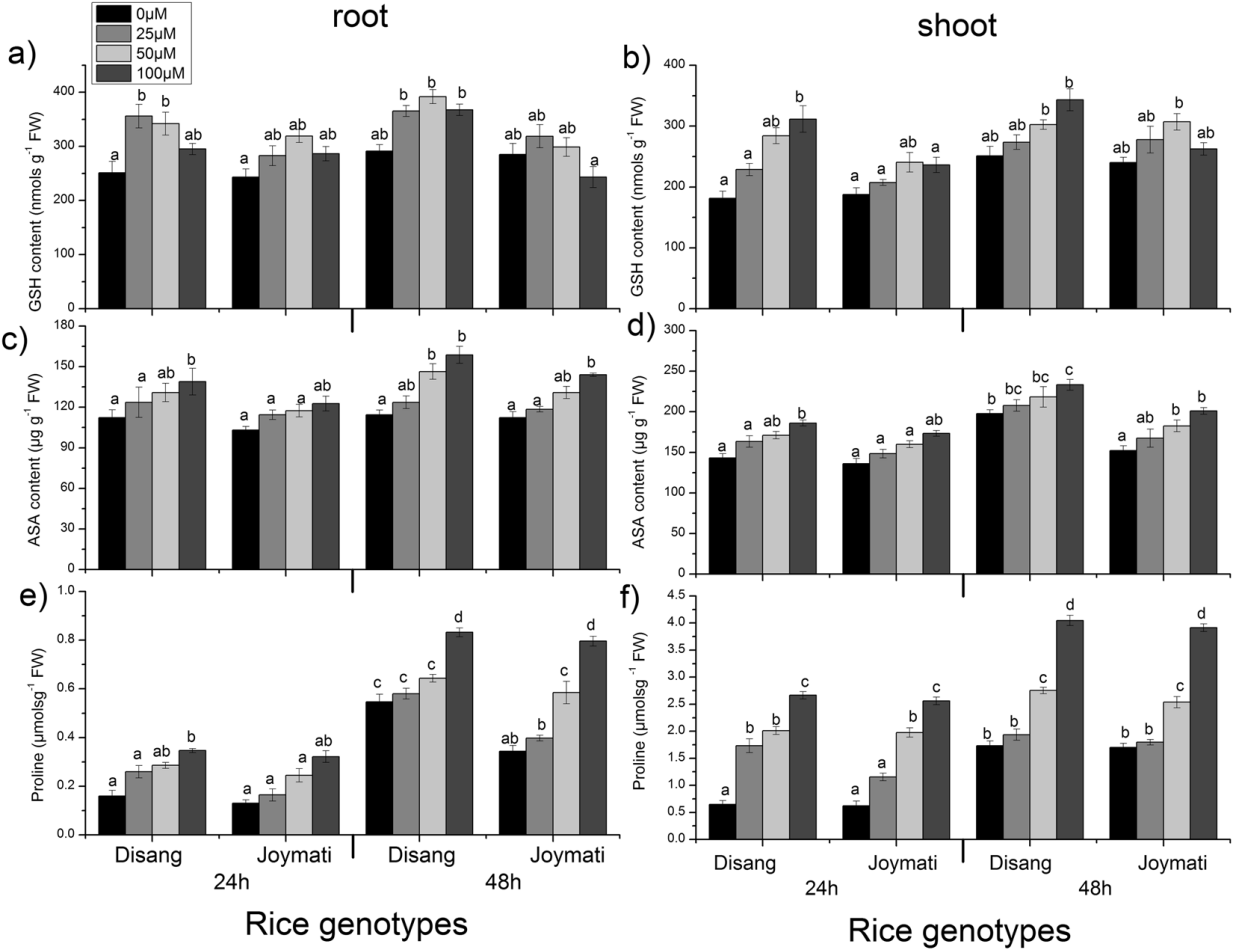
Effects of Al, on the Glutathione, Ascorbate and proline content in the roots (**a**,**c**,**e**) and in the shoot (**b**,**d**,**f**) of the two rice varieties. Seedlings were exposed to 0, 25, 50 and 100 µM AlCl_3_, containing 0.5 mM CaCl_2_ (pH 4.5) for 24 h and 48 h. Values are mean ± SE (n = 3) of three separate experiments. Means denoted by the same letter were not significantly different at P < 0.05 according to Tukey multiple range test.

Contents of non -enzymatic antioxidants such as glutathione (GSH), ascorbic acid (AsA) and proline displayed an uptrend on Al treatment in Disang and Joymati cultivars. Both cultivars showed a significant enhancement in GSH accumulation when the seedlings were treated with Al. But the increase was significantly higher in Disang when compared to Joymati with the exception at 100 µM; root for both the cultivars and shoot for Disang, where there was a decrease (Fig. 7a,b). For AsA there was all throughout increase in concentration for both cultivars but the increase was profound in Disang. Proline is an osmoprotectant cum non-enzymatic antioxidant, whose content was observed to increase in root and shoot with respect to Al dose (Fig. 7c,d). Similar to GSH and AsA increase was more significant in Disang (Fig. 7e,f).

#### Organic Acid exudates measurement

**Figure 8:**
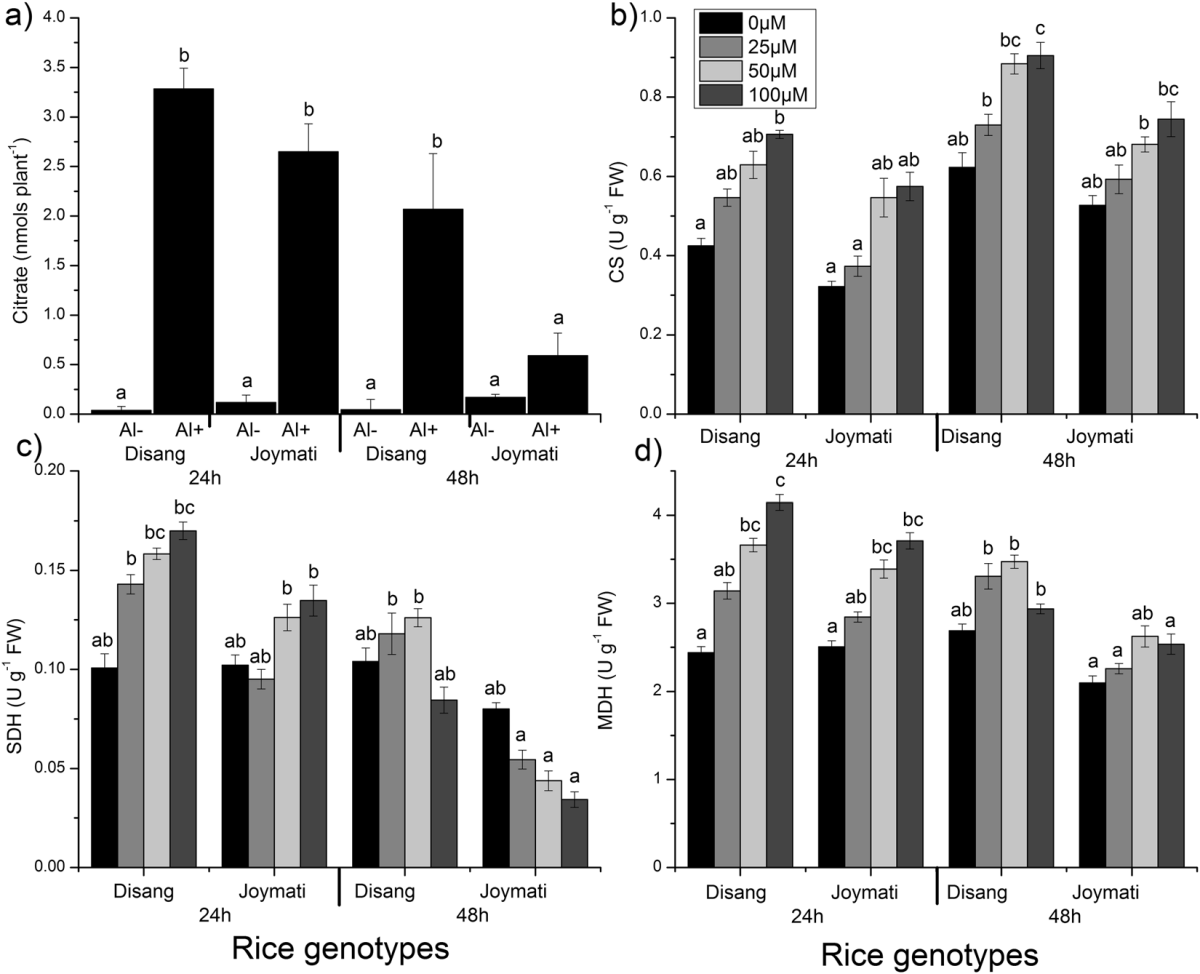
Effects of Al, on organic acid and metabolite enzyme. Citrate exudation as affected by the Al (0 and 100 µM AlCl_3_) treatment in rice genotypes at 24 h and 48 h (**a**). The activity of the citrate synthase (**b**) succinate dehydrogenase (**c**) and malate dehydrogenase (**d**) and organic acid content in the roots of the two rice varieties. Seedlings were exposed to 0, 25, 50, 100 µM AlCl_3_, containing 0.5 mM CaCl_2_ (pH 4.5) for 48 h. Values are mean ± SE (n = 3) of three separate experiments. Means denoted by the same letter were not significantly different at P < 0.05 according to Tukey multiple range test.

Among malate and citrate, later was observed to be the major organic acid (OA) exudate on exposure to Al stress in *Indica* rice cultivars (Figs 8a, S8). In stress condition citrate content was observed to be more in tolerant variety when compared to sensitive (Fig. 8a).

#### Organic acid synthesizing enzyme assay on Al stress exposure

Enzymes responsible for organic acid metabolism like, citrate synthase (CS), succinate dehydrogenase (SDH) and malate dehydrogenase (MDH) were studied. CS activity was detected to undergo enhancement on exposure to stress in both cultivars (Fig. 8b). SDH activity increased in Disang throughout, while in Joymati, activity slightly increased after 24 h but decreased at 48 h interval (Fig. 8c). MDH activity increased in stress condition for both varieties. The increase for Disang was profound when compared to Joymati (Fig. 8d).

#### Measurement of NAD^+^-NADH and its ratio on Al stress

**Figure 9:**
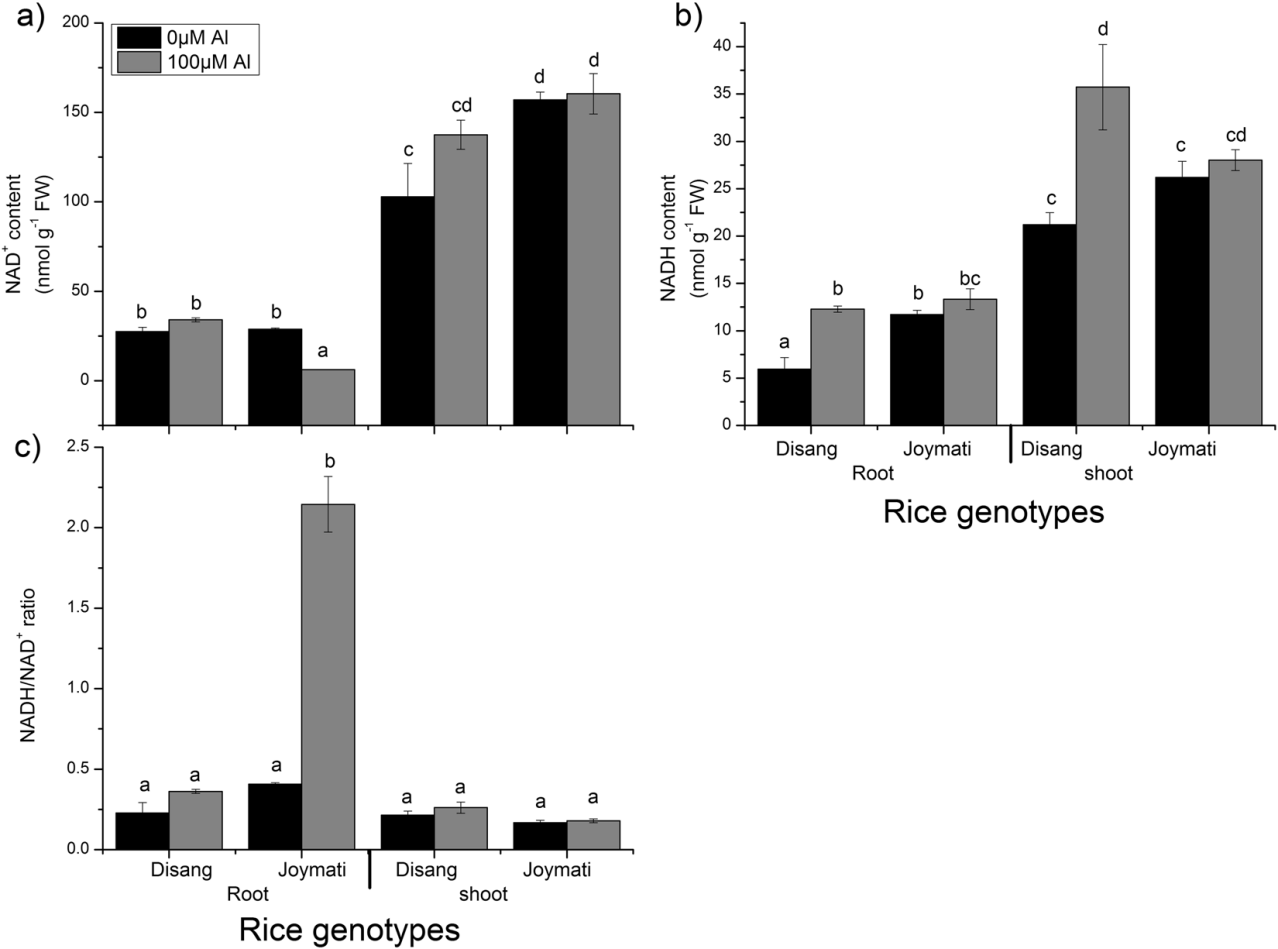
NAD^+^, NADH content and its ratio of rice plants under Al stress. Absolute quantification of NAD^+^, NADH by microtiter plate reader coupled enzyme assay in different replication (**a**,**b**) and ratio of NADH-NAD^+^. (**c**) Results simply showed the difference between control and treated root tissue of rice plant after 48 h exposure of 0 and 100 µM Al concentration Means denoted by the same letter were not significantly different at P < 0.05 according to Tukey multiple range test.

The content of NAD^+^ and NADH and its ratio in course of Al stress of 100 µM and untreated conditions was analyzed. The content was calculated from the standard graph of NAD^+^ and NADH. It was observed that the NAD^+^, NADH and its ratio increased under Al stress. The NAD^+^ content increased in stressed condition at 48 h in Disang while in Joymati it decreased in root sample. The shoot sample showed increased NAD^+^ content in both cultivars. NADH content also increased in Disang and Joymati varieties in root and shoot sample but the increase was profound in Disang. NADH and NAD^+^ ratio didn’t show significant difference within treatments for each cultivar, but in Joymati, significantly difference was observed in root sample (Fig. 9a–c).

#### Analysis of metabolites through LC-MS

The HPLC chromatogram of both varieties were scored for change in each peak area, at 254 nm. The peaks with significant difference in area between the two varieties were processed for mass chromatography. From MS, stronger signals were selected for MS^2^ analysis. MS^2^ signals having m/z 195, m/z 243, m/z 275, m/z 290 and m/z 540 were searched for in the mass bank database^12^ and the compound Gluconic acid, Uridine, L-Saccharopine, Catechin were identified whereas one compound remains unknown (Table S2).

#### Isozyme study of antioxidants enzymes

To inquire into the consequences of Al stress on antioxidant isoenzymes, the native PAGE profile for antioxidant enzymes isoforms was performed. The patterns of iso-enzymes of superoxide dismutase (SOD), ascorbate peroxidase (APX), guaiacol peroxidase (POX), glutathione reductase (GR) and catalase (CAT) in root and shoot were analyzed. The content of iso-enzymes also showed alteration in response to different concentrations of Al. In root four SOD, four POX, one APX two GR and one CAT iso-enzyme were detected, in stress and control condition. When in stress, SOD and POX content was slightly in decline while GR, APX and CAT remain unchanged (Fig. S9). Whereas in shoot, three SOD, three POX, one APX two GR and two CAT iso-enzyme were detected. In stress condition, all isozymes showed uniformity except for SOD which showed decline at 25 and 50 µM (Fig. S9).

#### Activity of ROS scavenging enzyme

**Figure 10:**
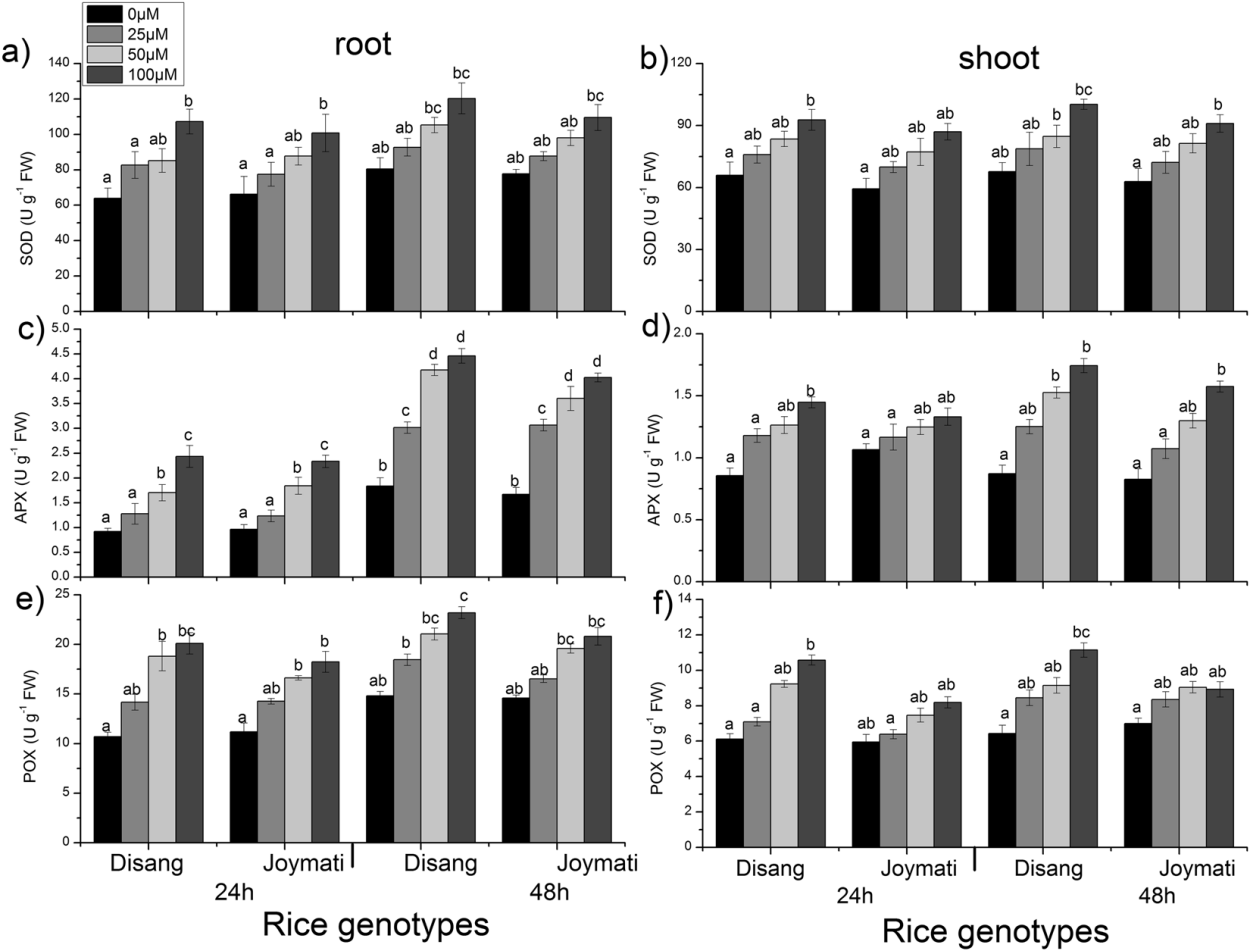
Effects of Al on the activity of SOD, APX and POX, in root (**a**,**c**,**e**) and shoot (**b**,**d**,**f**) of the two rice varieties. Seedlings were exposed to 0.5 mM CaCl_2_ (pH 4.5) containing 0, 25, 50, 100 µM AlCl_3_ for 48 h. Values are mean ± SE (n = 3) of three separate experiments. Means denoted by the same letter were not significantly different at P < 0.05 according to Tukey multiple range test.

Al stress significantly increased the activities of SOD, CAT and POX in root and shoot tissue. In root, increase in SOD activity was detected as the dose of Al increased. In root, higher SOD activity was detected at Al dose of 100 µM after 24 h of exposure. After 48 h of stress, a gradual increase of SOD activity was detected as the dose of Al was enhanced (Fig. 10a). Similarly, in shoot, (Fig. 10b) significantly elevated SOD level was observed as compared to control. APX activity on Al stress exposure, was observed to get elevated significantly in both root and shoot tissues, (Fig. 10c,d) after 24 and 48 h. POX activity was also examined in both root and shoot. In both tissues, significant gradual increment of POX activity was noted with the increase of Al dose (Fig. 10e,f).

**Figure 11:**
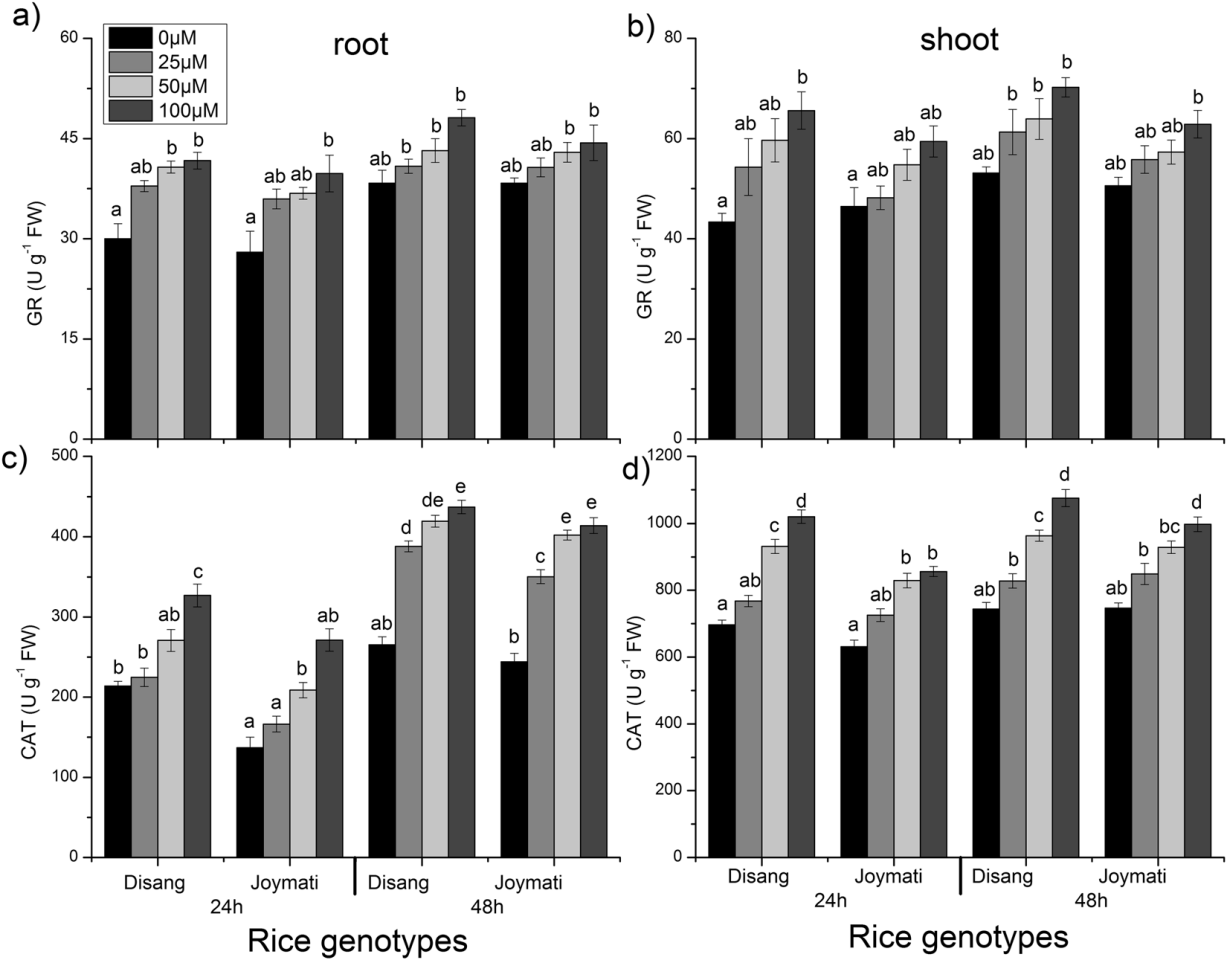
Effects of Al on the activity of GR, CAT in root (**a**,**c**) and shoot (**b**,**d**) of the two rice varieties. Seedlings were exposed to 0.5 mM CaCl_2_ (pH 4.5) containing 0, 25, 50,100 µM AlCl_3_ for 48 h. Values are mean ± SE (n = 3) of three separate experiments. Means denoted by the same letter were not significantly different at P < 0.05 according to Duncan’s multiple range test.

**Figure 12:**
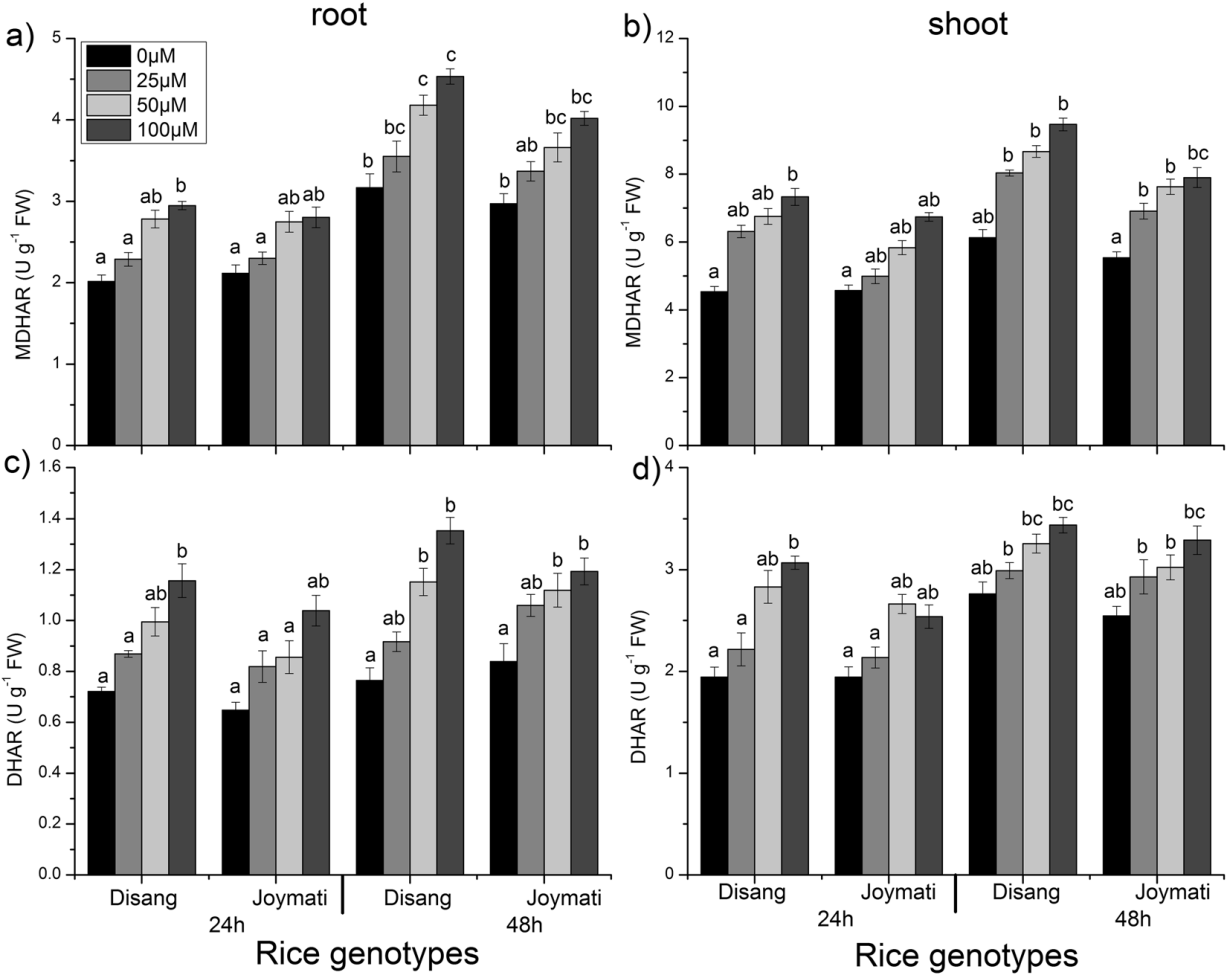
Effects of Al on the activity of MDHAR, DHAR in root (**a**,**c**) and shoot (**b**,**d**) of the two rice varieties. Seedlings were exposed to 0.5 mM CaCl_2_ (pH 4.5) containing 0, 25, 50,100 µM AlCl_3_ for 48 h. Values are mean ± SE (n = 3) of three separate experiments. Means denoted by the same letter were not significantly different at P < 0.05 according to Duncan’s multiple range test.

Under Al stress, significantly enhanced GR activity was observed in the rice. GR activity was found to enhance in a time and dose dependent way, in both root and shoot (Fig. 11a,b). The CAT activity in treated root was raised as compared to control and amidst all the doses it was more in 100 µM Al treated root (Fig. 11c,d). However, in shoot after 24, 48 h of treatment it was detected that the activity was in increasing order with the increase in Al concentration. Monodehydroascorbate reductase (MDHAR) content also increased significantly after 24 and 48 h of stress (Fig. 12a,b). Dehydroascorbate reductase (DHAR) activity as determined in response to Al stress, was observed to be elevated for treated samples in comparison to control check. In both tissue samples, gradual increase of DHAR activity was observed with increment of Al dose (Fig. 12c,d). Disang showed profound APX, GR, MDHAR and DHAR activity in comparison to Joymati under stressful condition.

#### Transcript abundance of Al stress responsive stakeholders

**Figure 13:**
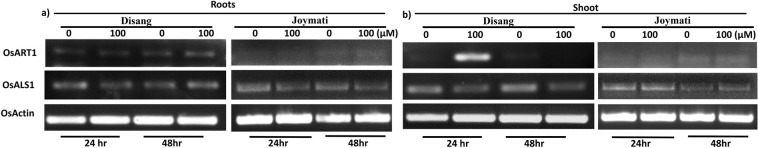
Al induced gene expression studies in Root (**a**) shoot (**b**) of Disang rice cultivar and Joymati rice cultivar under Al toxicity at 24 and 48 h intervals.

**Figure 14:**
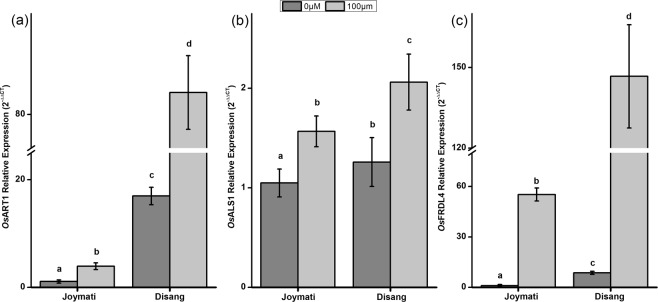
Relative expression (2^−ΔΔCT^) of Al induced genes in root. (**a**) *Os*ART1, (**b**) *Os*ALS1 and (**c**) *Os*FRDL4. Seedlings were exposed to 0.5 mM CaCl_2_ (pH 4.5) containing 0, 100 µM AlCl_3_ for 48 h. Values are mean ± SE (n = 3) of three separate experiments. Means denoted by the same letter were not significantly different at P < 0.05 according to Duncan’s multiple range test.

Al induced gene expression studies were done in both Disang and Joymati cultivar. *Os*ALS1didn’t show much difference (0.3 times) in expression in both the cultivars under Al stress; whereas, for *Os*ART1 expression was negligible (Disang showed 21.3 times more expression) in the sensitive variety, Joymati. (Figs 13, 14). The semi-quantitative PCR was further supported by real-time PCR. *Os*FRDL4, the citrate transporter showed higher expression (by 1.6 times) in the tolerant cultivar Disang under the influence of Al stress (Fig. 14).

### Interaction of Al^3+^ with other stake holders during stress response

#### Impact of Al-Mn interaction on growth physiology

Al-Mn interaction assay was conducted and it was observed that the Mn toxicity caused more damage in Joymati variety when compared to Disang variety. The leads pointed that Al addition relieved the toxic effects of Mn on rice growth but not significantly (Fig. S10). The figures of the seedlings in various doses demonstrate the slight alleviation of Al on Mn toxicity (Fig. 14).

#### Impact of Al-GSH interaction on growth physiology

**Figure 15:**
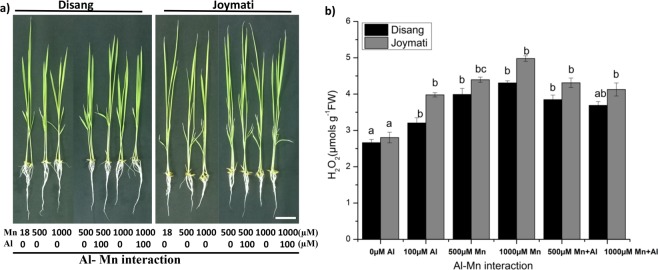
Effects of Al-Mn interaction in rice plants. Effect of Al on rice under Mn toxicity in a long-term experiment. Toxicity symptoms of Mn toxicity and ameliorating effect of Al on rice (**a**) and H_2_O_2_ content measurement in root sample (**b**). Rice seedlings (6 days old) were grown in nutrient solution with 18, 500, 1000 µM Mn in the presence or absence of 100 µM Al for 5 day. Values are mean ± SE (n = 3) of three separate experiments. Means denoted by the same letter were not significantly different at P < 0.05 according to Duncan’s multiple range test.

Al-GSH interaction study revealed that GSH has a crucial role to play in the amelioration and reduction of Al toxicity in both rice variety, especially in case of the sensitive variety Joymati where marked differences was observed. There was marked increase in root length when GSH was exogenously given, whereas no such observations were made on shoot length (Fig. S11). Similar trend was followed when observed for H_2_O_2_ content, which showed drastic reduction in root H_2_O_2_ content (Figs 15, S11).

## Discussion

Rice is an important cereal consumed by a very large segment of world population. Though rice is comparatively tolerant to the threat of acid soil it’s production is highly constrained. A large portion of the soil of North East India is highly acidic, yet it has rich diversity of *Indica* rice varieties. Our long term goal is to identify any new knowledge that can be gathered through study of these varieties. The exhaustive screening and the toxicity of Al stress in combination with acid soil has been documented in our earlier report^4^. In the study, random 22 varieties of North East India were screened physiologically for Al stress tolerance along with two previously reported tolerant and sensitive varieties. The present report was carried out to show the differential redox, metabolic and physiological fingerprint of two contrasting cultivars native to NE India, Disang and Joymati (identified in the earlier report) under Al stress at acidic pH.

Root growth, the initial and potent marker for Al stress in acidic condition was significantly inhibited in the two varieties (Fig. 1). The phytotoxic symptoms detected were very much alike to those described earlier^16,17^. Shoot length and, root: shoot ratio showed significant changes under Al stress at low pH (Fig. S1). The extent of oxidative stress was estimated by determining ROS accumulation, H_2_O_2_ and O_2_^−^ content. In Joymati, total ROS accumulation was higher on exposure to Al stress at low pH, when compared to Disang (Fig. 2). In maize and pea plant Al-induced ROS production caused excess cell rigidity^5^. Our histochemical observations corroborated with the quantitative studies^4^. ROS is generated due to cellular metabolic activities in plants, generally a homeostasis is maintained between the ROS generating and scavenging machinery but when in stress the homeostasis stands disturbed^9^. A significant increment of H_2_O_2_ and O_2_^−^ upon Al treatment was observed when compared to control in plant tissues (Fig. 3a–d). Though, ROS works as signalling molecules to activate the response mechanism leading to tolerance, but on excess accumulation the entire machinery succumbs. The high amount of ROS causes stomata closure and decrease in photosynthetic rate resulting in hampered crop productivity^18^. As loss of ROS homeostasis have its direct effect on photosynthetic apparatus, different stakeholders were analysed to have clear picture of the consequence of Al stress on photosynthesis. Diminution in total chlorophyll content (Fig. 4a); the photosynthetic pigments *viz*., chlorophyll a, chlorophyll b and carotenoids content in response to Al stress after 48 h (Fig. S2) was observed when exposed to higher dose of Al at low pH. Though the preliminary site of injury is root but the effect can be felt at the whole plant level^18^. Al stress also led to decrease in stomatal conductance, depending on dose (Fig. 4b). Aluminum induced decrease of stomatal conductance in *Citrus limonia*^19^, *Cleoptara tangerine*^20^, Sour pumelo^21^ and in rye^22^ has been reported previously. These decrease in pigment content and stomatal conductance poses a negative effect on the whole photosynthetic machinery.

Analysis of photosynthetic efficiency through florescence measurements depicted drastic decrease on exposure to Al. The correlation analysis was performed for all the chlorophyll fluorescence parameters (Fo, Fm, Fv/Fm, Fo′, Fm′, Fv′/Fm′, Y(II), qP, qN, qL, NPQ, Y(NO), Y(NPQ) and ETR). The Fv/Fm values being a robust indicator, are generally very coherent within varieties and individual plants; as such, any modest decline is well detectable and signifies clearly that loss of viability in stress condition. Broadly, Fv/Fm, Fv′/Fm′, Y(II), and qP have been named photochemical quenching parameters, and NPQ is a nonphotochemical quenching functions^23^. The Non-Photochemical Quenching (NPQ) of chlorophyll fluorescence is the most effective photoprotective reaction in plants. The study observes that the NPQ, Y(NPQ) increased while Y(NO) decreased under Al stress condition (Table S1). For the very first time, photosynthetic efficiency of two contrasting varieties of rice to Al stress under acidic condition was studied.

Consequence of exuberant ROS induced oxidative damage was documented by measuring carbonyl content in both root and shoot. After Al stress imposed, an heightened level of protein carbonyl content was detected which is actually protein oxidation promoted by ROS (Fig. S3). Protein carbonyl content is proposed to be an indicant for oxidative damage^24,25^. LOX mediates membrane lipid peroxidation in presence of excess ROS which finally directs to membrane damage and cell death. LOX activity and MDA content was observed to increase in time and dose dependent way on exposure to Al stress at low pH (Fig. S3).

Plant in its attempt to restrict the damage due to environmental cues and isolate the affected cells resorts to callose deposition. Deposition and assemblage of callose reverberates physiological stress as well as the level of cumulative cell injury, particularly in the Al^3+^-sensitive cultivars^16^. Significant changes in callose accumulation was observed in sensitive cultivar under higher concentration of the Al stress while in Disang cultivar accumulation was less (Fig. 6). Similar response was observed earlier in the two *Japonica* cultivars of rice^16^, after 60 h exposure to 80 µM Al. Besides isolating the damaged cells plants also responds by providing structural rigidity through lignin deposition^26^. Heightened lignification under biotic and abiotic stress can assist as a roadblock restricting the entry of metals and pathogens into tissue. Previous studies also described that Al caused the assemblage of lignin in roots of *Indica* rice^27^ and wheat^28^.

GSH, AsA and proline are involved in physiological and stress related defence mechanisms in plants by providing redox homeostasis. Schutzendubel^29^ observed that ROS accumulation is favoured by lack of GSH content and results in disintegration of developmental processes. In this study, Disang showed more GSH accumulation than Joymati (Fig. 7a,b). GSH is needed in detoxification of oxidative stress caused by heavy metals in plants^30^. Increased GSH level was detected in pea root system in response to Al stress while in shoot it was reverse^3^. AsA interacts with enzyme activities and reduces cellular damage caused by ROS molecules through synergistic activity with other antioxidants^9^. Less accumulation of AsA content as equated to control for root was observed (Fig. 7c,d). The results also suggest the deposition of proline in response to Al stress in root and shoot (Fig. 7e,f). Proline deposition precludes membrane deformation; act as a hydroxyl radical scavenger, and an osmoprotectant^31^. Similar results were observed in rice^32^, maize^33^, and *Borago officinalis*^34^.

NADH-NAD^+^ content helps in having a clear view of the redox status of a cell when exposed to sub-optimal environments. The NADH-NAD^+^ content is being depicted for the first time in response to Al stress. Determination of NADH-NAD^+^ content revealed higher prevalence of NAD^+^ (oxidizing agent) in root and shoot sample of Disang when compared to Joymati depicting better redox status. Similar revelations were made in *Arabidopsis* treated with quinolinic acid^35^ and *Atgpdhc1* mutant line^36^; in pea under water deficit^37^ and in tobacco *nad7* mutant^38^. NADH content was also higher as compared to control. Whereas the NADH/NAD^+^ ratio was not significantly higher in root and shoot sample under Al stress in Disang (Fig. 9) similar to that of pea under light and CO_2_ stress^39^.

Antioxidative enzymes such as superoxide dismutase (SOD), ascorbate peroxidase (APX), and catalase (CAT) serves as important ROS-scavenging enzymes under Al toxicity^40^. Hence analysis of the isoforms is of utmost importance to have a glimpse of the overall machinery. As the analysis revealed four distinct bands for SOD in root and three band in shoot tissues, indicating that these SOD isoforms are expressed under Al stress (Fig. S9). Plants under Al stress showed variation in SOD isoform’s band intensity in 25 and 50 µM Al treatment in shoot. The root and shoot samples showed only one prominent APX isoenzyme band in response to different concentration of Al^3+^, and the band intensity slightly differs from that induced due to higher concentration of Al. Similar results were reported in rice earlier under Al stress^40^. GR isoforms in both root and shoot detected two isoform bands in both root and shoot tissue samples. CAT isoenzyme band pattern showed significantly increased band intensity in root. Between the cultivars, although distinct differences were not observed still in case of POX and APX increase in band intensity with Al dose in Disang and *vice versa* in Joymati can be noted.

SOD, APX, POX, GR, CAT, MDHAR, DHAR antioxidant enzymes activity were also analysed, for their activity as ROS scavengers. It was observed that under Al stress condition SOD activity increased in root and shoot (Figs 10–12). SOD consists of different metalloenzymes *viz*., Cu-Zn SOD, MnSOD and FeSOD isoforms. SOD is involved in catalysing the reaction of dismutation of ROS molecules (O_2_^−^ to H_2_O_2_ and O_2_). Enhanced SOD activity was reported in response to Al stress in *Allium cepa*^24^. Previous reports also support the findings regarding increased SOD activity in response to Al stress in soyabean, maize and barley^6,41,42^. The intensity of SOD isoforms found to be increased under stressful conditions, which indicates its involvement in defence mechanism against oxidative stress condition in cytosol, mitochondria and chloroplast^43^. The induced synthesis of toxic free radicals, in response to abiotic stress, such as O_2_^−^ and H_2_O_2_ can be scavenged by APX, CAT, and POX antioxidant activity^44^ in plants. APX reduces H_2_O_2_ to H_2_O molecule by using ascorbic acid as an electron donor and this step is the initial step of ascorbate-glutathione cycle. APX activity was observed to be increased under Al stress in rice. Similar result was reported in rice under 160 µM Al stress^40^. It was suggested that APX has major role in scavenging H_2_O_2_ molecule under stress condition^44^. POX is another antioxidant enzyme involved in scavenging of ROS molecules and thus protect from oxidative injury^3^. By oxidizing phenolic compounds POX catalyses H_2_O_2_ degradation. In this study increased POX activity was observed in rice plants under Al stress in root-shoot tissues (Fig. 10e,f). Similar result were earlier observed in maize, pea and rice in response to Al stress^3,6,40^. Glutathione reductase is one of the key enzymes of the ascorbate -glutathione cycle that protects cells from oxidative damage and holds a high GSH/GSSG ratio encouraging cellular constancy and integrity^7^. We have also investigated GR activity, another enzyme of ascorbate-glutathione cycle, which also showed increased activity under Al stress in rice (Fig. 11a,b). We observed time dependent enhancement of GR activity under Al stress. Excessive ROS produced under stressed condition is finally scavenged by CAT activity. CAT activity in rice root samples treated with 40 µM Al was significantly enhanced in tolerant rice cultivar under Al stress^44^. The main purpose of MDHAR in Ascorbate glutathione cycle is to regulate the level of MDHA radical involved in non-enzymatic disproportionation and generation of DHA. MDHAR activity in treated samples increased in dose dependent fashion (Fig. 12). Higher MDHAR activity can regulate the MDHA content by reducing it into ASA by using NADPH^45^. DHAR contributes in ascorbate production by reducing itself with glutathione as reductant. Further ascorbate is utilized by APX to detoxify H_2_O_2_ to H_2_O. In this work, elevated DHAR activity was observed in treated root samples. DHAR activity was induced by Al after 24and 48 h of stress (Fig. 12c,d). DHAR activity has earlier been reported to increase at early stage of copper stress in *Arabidopsis*^46^.

Organic acid efflux due to Al stress in rice is somewhat controversial. Few reports suggest no significant role of organic acids towards aluminum tolerance^31^ whereas others report their active role^47^. In this case, when citrate exudation was studied, marked influence was observed on both sensitive and tolerant varieties in a time dependent fashion (Fig. 8a). The enhanced citrate exudation, observed on exposure to Al toxicity, leading to detoxification of Al by formation of OA–Al complexes and in the process preventing its uptake by root cells in the rhizosphere. The excess citrate exudation was confirmed by observance of increased abundance of *Os*FRDL4 transcripts (a MATE family citrate transporter, Fig. 14).

Organic acid metabolizing enzymes such as citrate synthase, malate dehydrogenase and succinate dehydrogenase were studied to compare their activities. Citrate synthase (CS) activity was found to increase under stress condition in both cultivars (Fig. 8b). Similar results were reported in rye^48^, *Plantago almogravensis*^49^ and soyabean^50^ on exposure to Al stress. CS is the rate limiting enzyme of TCA cycle and is responsible for formation of citrate, which is used in Al detoxification mechanism^51^. Succinate dehydrogenase (SDH, EC 1.3.5.1) activity increased in Disang variety, while in Joymati, SDH activity increased slightly after 24 h (Fig. 8c). SDH is the only enzyme of the Krebs cycle that is bound to the inner mitochondrial membrane and acts in the transfer of electron from succinate to ubiquinone^52^. On drought stress, SDH was found to be upregulated in *Ilex paraguariensis* leaves^53^. In summer months, SDH activity of cauliflower was observed to be less^54^; similarly, in cowpea on Al stress it showed decreased activity^40,55^. SDH, frequently denoted to as complex II, has a dual activity; hence plays a substantial part in both the tricarboxylic acid cycle and the aerobic respiratory chain by catalyzing the oxidation of succinate to fumarate, and the reduction of ubiquinone to ubiquinol, respectively. Malate dehydrogenase (MDH) activity increased under stress condition in both the varieties; activity was more in Disang compared to Joymati (Fig. 8d). The enzyme MDH catalyses the reversible reduction of oxaloacetate to malate and is crucial in multiple metabolic pathway. It is taking part as a central enzyme in the citric acid cycle that catalyzes the oxidation of malate. MDH activity was reported to be significantly increased in triticale^56^, plantago species^49^, and *Lupines albus* root^57^. MDH activity was also reported high in tolerant rice variety under salt stress^58^. Increase MDH activity in the *Halimione portulacoides* leaves when exposed to salinity and in *Casuarina equisetifolia* under drought^59^ has been described. While its differential expression was observed during high and low temperature stresses^60^.

Pot stress assay revealed more damage and discoloration in sensitive variety (Fig. S5). Total protein content in root was observed to increase with time and concentration of Al^3+^ hinting towards Al induced protein synthesis in root (Fig. S6a). However, in shoot, total protein content declined under Al stress (Figs S6, S7). In contrast, Roshani *et al*. found increased protein activity after 15 day of Al stress in rice seedlings^24^. Similar result was reported in kidney bean^61^. This enhancement in protein content might be due to synthesis of stress responsive stakeholders. Heavy metal stress including Al stress, induces proteins of 20 different functional classes and most of these include antioxidant defence elements. Further the change in abundance of proteins also depends on stress dose and duration^62,63^.

*Os*ART1 showed more expression in Disang compared to Joymati (Fig 13, 14). It plays a vital part in Al tolerance in rice by regulating the downstream genes responsible for both internal and external detoxifications of Al^12^. *Os*ALS1 is a half ABC transporter localized in vacuolar membranes and is responsible for sequestration of chelated Al in vacuoles^13^. Vacuolar sequestration forms an important and integral tolerance mechanism in rice^63^. ALS1 expression also showed significant changes on exposure to Al. *Os*FRDL4 is responsible for citrate exudation in the rhizosphere and was found to be more abundant in the tolerant variety Disang. MATE group citrate transporter has earlier been reported to provide Al tolerance in many crops^64,65^.

Al-Mn interaction assay was also conducted and it was observed that Mn toxicity hampered Joymati variety more as compared to Disang. The study revealed that the Al alleviates Mn toxicity minutely in both doses (500, 1000 µM) of Mn with 100 µM Al (Fig. 15). In the absence of Al, Mn significantly decreases root length. However, addition of Al markedly attenuated this suppression (Fig. 14). The morphology of the seedlings under several treatments demonstrate the attenuating effects of Al on Mn toxicity^66^. Due to Al toxicity rice leaves were observed to show orange yellow interveinal chlorosis, leaf tip death, poor growth and stunted growth^67^.

**Figure 16:**
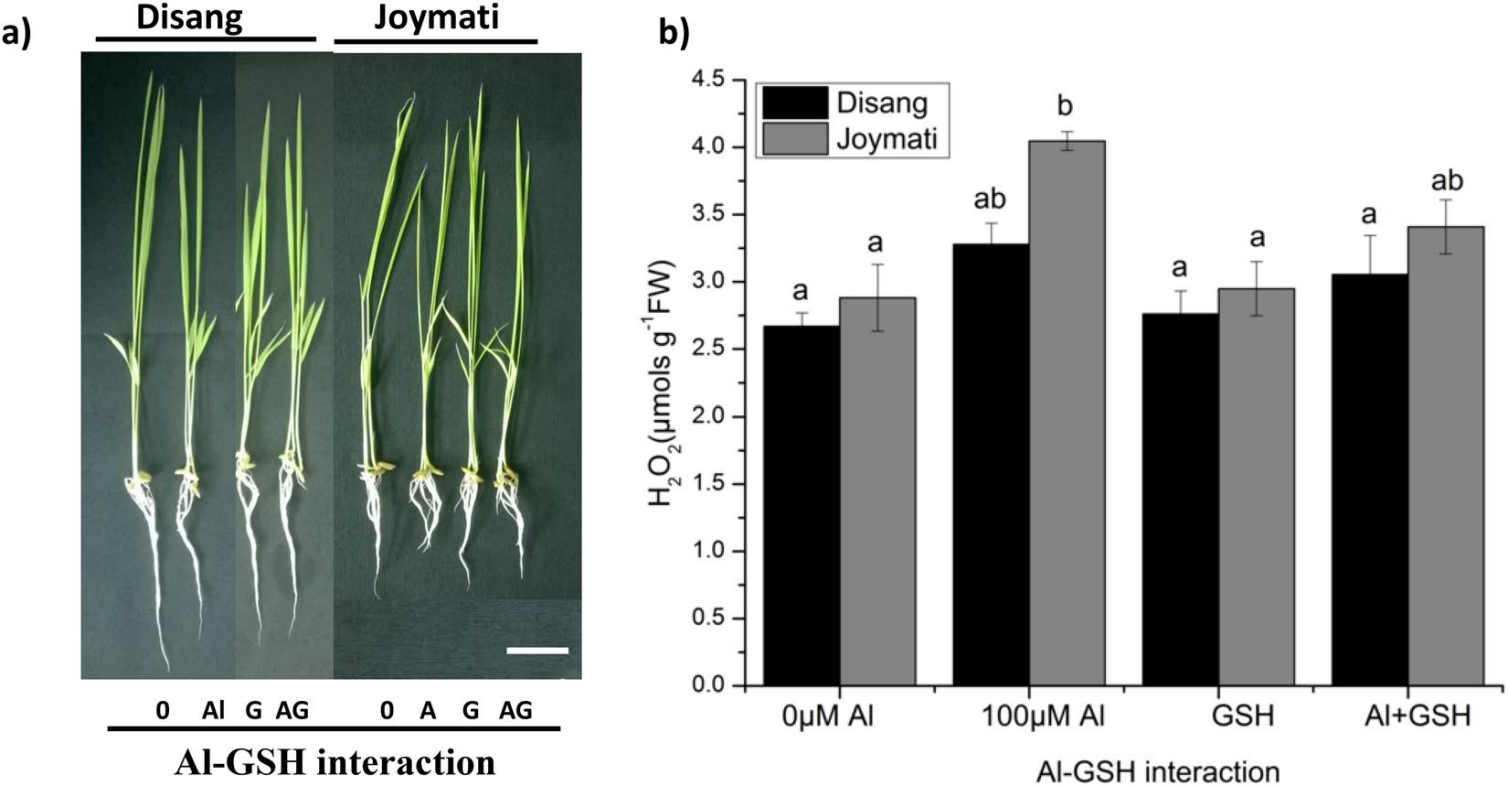
Effects of Al-GSH on rice under Al toxicity. GSH ameliorate the effect of Al on rice (**a**) and H_2_O_2_ content measurement in root sample (**b**). Rice seedlings (6 days old) were grown in nutrient solution with 0,100 µM Al and 1 mM GSH in the presence or absence of GSH for 48 h. Values are mean ± SE (n = 3) of three separate experiments. Means denoted by the same letter were not significantly different at P < 0.05 according to Duncan’s multiple range test.

Al-GSH interaction study revealed that the GSH has important role to play in the amelioration and reduction of Al toxicity in both rice varieties (Disang and Joymati). Root reduction was higher in Joymati as compared to Disang at higher concentration of Al (100 µM). This study thus revealed that the GSH is vital for Al detoxification (Fig. 16) in rice seedling^44^.

**Figure 17:**
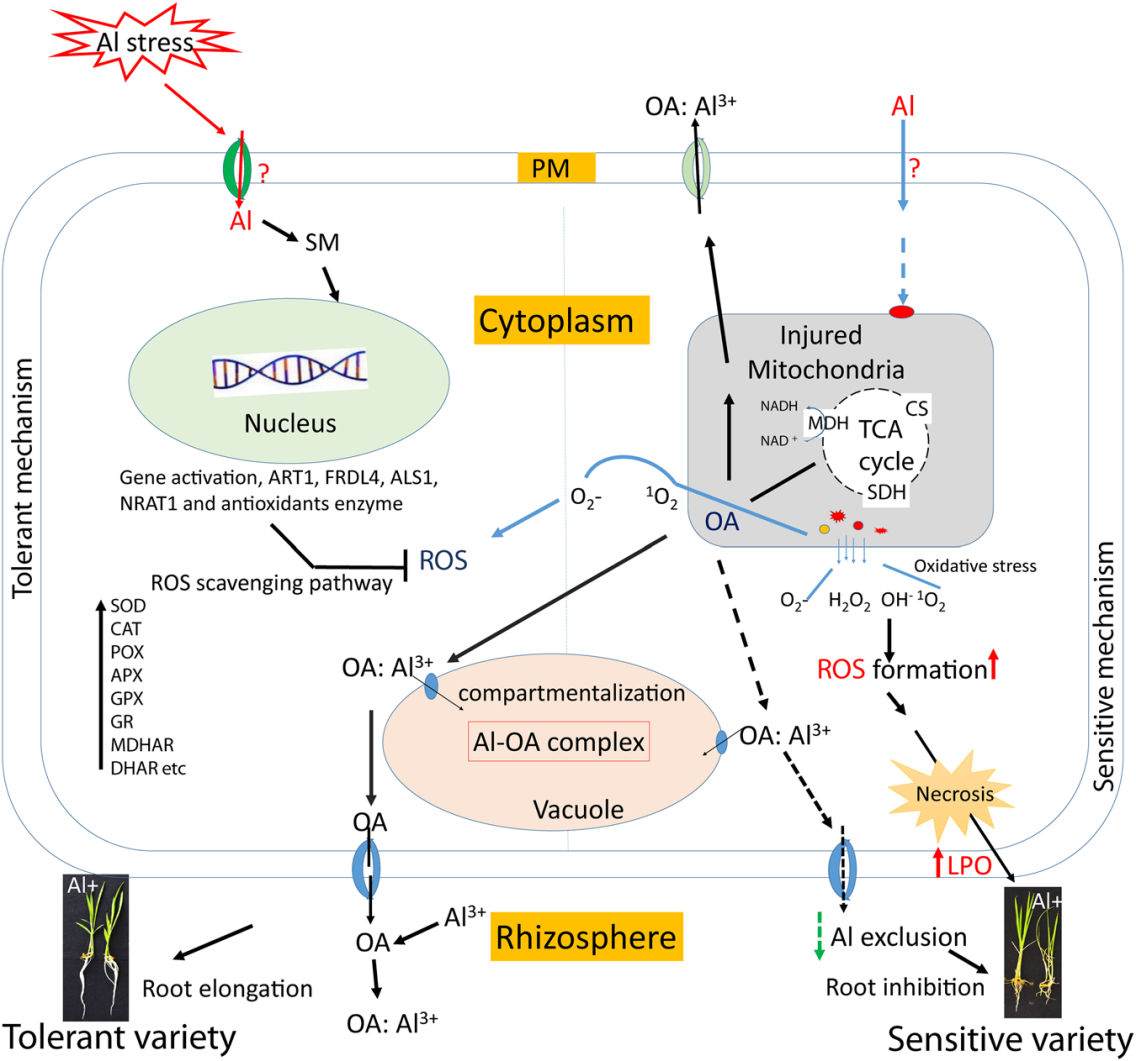
Mechanism of Al response in tolerant and sensitive *Indica* rice cultivars.

Based on the experiments an Al stress response mechanism of both tolerant and sensitive cultivar, has been concluded upon (Fig. 17). Al enters into the cell via endocytosis, interacts with cellular macromolecules and in the process disrupts ROS homeostasis^68^. Also it activates transcription machinery to code mRNA for organic acid synthesis and antioxidant enzymes. After the synthesis of organic acids Al^3+^ forms a complex, OA:Al^3+^ and gets deposited into the vacuole. Organic acid molecules also effluxed from root system into the rhizosphere to prevent further intake of Al by root cells. ROS scavenging enzymes and TCA cycle organic acid metabolizing enzymes activity increase and gets involved in detoxification of ROS molecules, in tolerant variety. In sensitive varieties, Al enters into the cell and interacts with the cellular molecules, rapidly enhancing ROS production without a strong antioxidant system. Organic acid is also secreted out in less amount. Lipid peroxidation was more and necrosis also occur in sensitive genotypes, which was responsible for the morphological changes in this cultivar.

There are several reports on rice showing differential responses of contrasting varieties to Al stress^2,28,44,69–72^. This report characterized two contrasting varieties to Al stress that have been screened out from the numerous varieties of North East India which is a hotspot of *indica* rice diversity^4^. Citrate was the major organic acid synthesized in response to Al stress in rice of North East India. Also was reported for the very first time NADH-NAD^+^ status in response to Al stress in the contrasting rice varieties. Increased citrate exudation and better anti-oxidative defence was mainly responsible for better Al tolerance in Disang compared to Joymati.

## Methods

### Plant materials and growth condition

Rice genotypes Al Tolerant (Disang) and sensitive (Joymati) were selected from our previous study^4^. Adequate amount of viable rice seeds was taken and surface sterilized with 0.1% HgCl_2_ solution, for 3–5 minutes with continuous shaking. After this, HgCl_2_ solution was removed, rinsed with distilled water for 2–3 times. Seeds were then soaked in water for 12 h, in petri plates with moistened filter paper and germinated at 28 ± 2 °C for 3 days. After three days, the healthy germinated seedlings with similar height of root and shoots were shifted into Planton box (400 ml; Tarsons, India) holding Hoagland nutrient medium. Seedlings were grown for a period of 5 days in a growth chamber under white light with photon flux density of 220 μmol m^−2^ s^−1^ (PAR) with 14 h photoperiod at 28 ± 3 °C, day: night temperatures. The relative humidity was controlled at 65 ± 5%. After every two days the medium was changed for healthy growth. On the sixth day rice seedlings were pretreated with 500 µM CaCl_2_ (pH 4.5). The very next day of pre-treatment, solution was discarded and the plants were treated with Al in the form of aluminum chloride (AlCl_3_) supplemented with 500 µM CaCl_2_ in a dose dependent (0, 25, 50 and 100 µM) and time dependent (24, 48 h) manner with a constant pH of 4.5.

### Oxidative stress in rice on Al^3+^ excess

#### ROS accumulation

ROS accumulation in root apex was labelled by using 2, 7-dichlorofluorescein diacetate (H_2_DCF-DA), a ROS-specific fluorescent probe, as described by Maffei *et al.*^73^. Briefly, root apex was incubated with 10 mM HEPES-NaOH buffer (pH 7.5) containing 10 μM H_2_DCF-DA for 30 min in dark. The root apexes were then washed with fresh buffer prior to detection using an epifluorescence microscope (Nikon, Tokyo, Japan).

#### Superoxide radical and hydrogen peroxide estimation

O_2_^−^ and H_2_O_2_ content was determined following the method of Elstner *et al*.^74^ and Sagisaka^75^ respectively.

### Damage due to oxidative stress in rice on Al^3+^ excess

#### Photosynthetic pigments and stomatal conductance of Al stressed seedlings

For photosynthetic pigment, 100 mg of fresh leaf was taken into a test tube holding 5 ml of DMSO. The test tube was then allowed to stand in an oven at 60 °C for about 4 h to ease the extraction of the pigments. After 4 h, the samples were cooled to room temperature and; absorbance was read at 454, 645 and 665 nm on spectrophotometer^76^. For the study of stomatal conductance randomly selected 10–15 leaves of each treatment and control were examined using a porometer (AP4 cycling porometer, Delta-T Devices, Cambridge, UK). The measurements were scored from the abaxial surface of the leaf between 10.00 h and 14.00 h. The readings were accomplished during one-hour to avoid the diurnal pattern of variation of the leaves.

#### Chlorophyll Fluorescence

Chlorophyll fluorescence measurements were performed using Junior PAM chlorophyll fluorimeter (Walz, Germany). For assessment of dark and light-adapted parameters, The following parameters were derived from the final measurements obtained after the 15 min dark adaptation and light adaptaion Fo, Fm, Fv/Fm, and Fo′, Fm′, Fv′/Fm′, Y(II), qP, qN, qL, NPQ, Y(NO), Y(NPQ) and ETR. All parameters were computed as defined previously^77^. Six measurements were obtained for each parameter.

#### Measurement of protein carbonylation and lipoxygenase (LOX)

For determination of protein oxidation or carbonylation, the carbonyl content of 1.0 g plant sample was scored following Verbeke *et al*.^78^.

LOX activity was estimated from 1.0 g of tissue by estimation of linoleate hydro peroxidation activity spectrophotometrically at 25 °C by monitoring the enhancement in absorbance at 234, due to the transition of linoleate into corresponding hydroperoxide^79^. An extinction coefficient of 25 mM^−1^ cm^−1^ was used to convert absorbance value to nmol of conjugated dienes (hydroperoxy linoleic acid).

#### Lipid peroxidation determination

For lipid peroxidation, MDA was quantified following the protocol of Heath and Packer^80^.

#### Callose accumulation

Al^3+^-induced callose accumulation was appraised by an aniline blue staining procedure following the protocol stated by Kauss^81^ and quantification was done adopting the procedure of Kauss *et al*.^82^.

#### Lignin accumulation

For lignin quantification from root segments Syros *et al*.^83^ protocol was adopted.

#### Pot stress assay

The rice seeds were surface-sterilized in 1% (v/v) sodium hypochlorite solution for 5 min, washed exhaustively with deionized water, and then allowed to wet in deionized water overnight. The seeds were germinated in an incubator at 30 °C for 3d, and then transferred to soil pot. The nutrient solution was provided every week. After 45 days of growth, pre-treatment with 0.5 mM CaCl_2_ (pH 4.5) for 1d was imparted, then; Al treatment (0, 100 µM) containing 0.5 mM CaCl_2_ with pH 4.5, was given at every 3d interval. After 15 days, the plants of both varieties were documented.

### Defence response of stressed rice seedlings to Al^3+^ stress

#### Estimation of soluble protein

Samples were prepared by homogenising 200 mg leaf and root tissue in 1.5 ml pre-chilled phosphate buffer (0.1 M, pH 7.0) containing 50 mg insoluble PVP. These were then centrifuged at 10,000 rpm at 4 °C for 15 min. The pellets were disposed and protein in the supernatant was quantitated with Bradford’s reagent. Each sample was diluted five times with extraction buffer before estimation. To 0.1 ml of this diluted extract, 1.5 ml Bradford’s reagent was added. Mixed thoroughly and absorbance was measured at 595 nm after 5 min. Standard curve was prepared using graded doses of bovine serum albumin (1 mg/ml) solution and protein profiling by SDS PAGE.

#### Glutathione, Ascorbate and Proline content

Total glutathione and ascorbate contents were determined by the method Griffith^84^ and Oser^85^, absorbance was recorded at 660 and 412 nm respectively. Whereas, for proline content, Bates *et al*.^86^ method was adopted.

#### Organic acid (citrate) exudation and measurement

In an aseptic environment, fifteen seedlings were grown on a plastic mesh floating over control growth solution containing 1% sucrose at pH 5.6. At day 5 after the seedlings have grown, they were shifted to separate wells of a 6-well plate containing 4 ml of control or Al-containing (100 µM Al) media. Both organic acid collection media were made by adding 1% (w/v) sucrose to the control growth solution, and the pH was maintained at 4.5. Rotary shaker was used to gently shake (40 rpm) the seedlings at 25 °C in the dark. Media were collected at 24 h and 48 h after transfer, each medium was measured for citrate content by a NAD/NADH cycle coupled enzymatic method^87^.

#### Organic acid synthesizing enzymes

The citrate synthase (CS, EC 4.1.3.7) activity was determined spectrophotometrically by measuring the reduction of acetyl coenzyme A with 5, 5-dithiobis (2-nitrobenzonic acid) at 412 nm for 3min^88^. Malate dehydrogenase activity (MDH, EC 1.1.1.37) activity was assayed as described by Gupta *et al*.^57^. Succinate dehydrogenase activity (SDH, EC 1.3.5.1) was determined by continuous recording of the reduction of DCPIP at 580 nm^89^.

#### Measurement of NAD^+^-NADH and its ratio on Al stress

The NAD^+^, NADH content were scored according to enzymatic reaction, for absorbance at 570 nm, content was calculated by using standards run concurrently with unknown^29,90^.

#### LC-MS sample preparation

Rice root sample (500 mg) were ground in 1.5 ml of deionized water. The mixture was then agitated in a water bath at 60 °C for 30 min and then centrifuged at 25 °C and 13,0000 rpm for 15 min. The supernatants were lyophilized and analyzed with UPLC-MS (Waters Xevo G2 Q TOF). Sample were injected into an ACQUITY bridged ethyl hybrid (BEH) C18 column (100 × 2.1 mm i.d., 1.7 µm, Waters). Column temperature was set at 35 °C. The mobile phase comprised of A (0.5% v/v formic acid in water) and B (100% CH_3_CN). The gradient for the mobile phase were as follows: 0 min, 99% A (held 5 min), 20 min, 40% A. the flow rate was 0.3 mL/min. UV detector wavelength was set at 254 nm. Data analyzed with Mass bank data base^91^.

#### Isozyme study of antioxidant enzymes

The extraction for the enzymes was done as suggested by Larkindale and Huang^92^. For superoxide dismutase (SOD), ascorbate peroxidase (APX), peroxidase (POX), glutathione reductase (GR) and catalase (CAT) isozyme study Rucinska *et al*.^93^, De Gara *et al*.^94^, Rao *et al*.^95^, Smith *et al*.^96^, Lee and Shin^97^ respectively; protocols were followed.

#### Antioxidant enzyme activity

After extraction of enzyme^85^, SOD (EC 1.15.1.1), activity was ascertained by measuring the suppression of photochemical reduction of NBT spectrophotometrically at 560 nm^57^.

For CAT (EC 1.11.1.6), activity estimation^98^, the alteration in absorbance was recorded at 240 nm. The enzyme activity was expressed as unit g^−1^ fresh weight.

For APX (EC 1.11.1.11) assay^99^, the decrease in absorbance was documented at 290 nm at 15 s intervals for 2 min.

POX (EC 1.11.1.7), activity was quantitated by calculating the oxidation of guaiacol^100^. The absorbance change was noted at 15 s interval for 2 min at 470 nm.

For estimation of GR (EC 1.11.1.9) activity^96^, change in absorbance at 412 nm was checked at 15 s interval upto 2 min.

MDHAR (EC 1.6.5.4), activity was assayed by documenting the oxidation of NADH at 340 nm^101^.

DHAR (EC 1.8.5.1), activity^102^ was recorded using an extinction coefficient of 14 mM^−1^cm^−1^ for ascorbate at 265 nm.

#### Organic acid transporter gene expression studies

To study the expression pattern of ART1, ALS1, FRDL4 and reference gene actin by semi-quantitative and quantitative real time PCR, seedlings of the two rice cultivars were treated with different aluminum doses (0 and 100 µM) for time periods of 24 h and 48 h. Whole roots and shoots tissue samples were excised and frozen in liquid nitrogen within 5 mins of harvest. Total RNA was extracted using the NucleoSpin RNA Plant (MACHEREY NAGEL). cDNA synthesis was done following manufacturer’s instructions using PrimeScript^TM^ first strand cDNA synthesis Kit (TAKARA Clontech). The primer sequences for semi-quantitative and quantitative real time PCR of *OsART1* were Fw 5′- CAGTGCTTCTCGTGGGTCTT-3′, Rv 5′- CCTGTGCGTGAAGAACCACT-3′^13^; *OsALS1* were Fw 5′-GGTCGTCAGTCTCTGCCTTGTC-3′, Rv 5′-CCTCCCCATCATTTTCATTTGT-3′^103^; *OsFRDL4* were Fw 5′-CAGTGCTTCTCGTGGGTCTT-3′, Rv 5′-CCTGTGCGTGAAGAACCACT-3′^104^ and *OsActin* were Fw 5′-GACTCTGGTGATGGTGTCAGC-3′, Rv 5′- GGCTGGAAGAGGACCTCAGG -3′ respectively.

### Interaction of Al^3+^ with other stakeholders during stress response

#### Impact of Al-Mn interaction on growth physiology

After 5 days of growth, the germinated seedlings were shifted to 0.5 mM CaCl_2_ (pH 4.5) for further 1 d. Finally, 6 d-old seedlings were used in experiments with various Al and Mn treatments. To discover a desirable Al dose for interaction studies, seedlings were allowed to grow in half strength Hoagland nutrient solution containing 0, 25, 50 or 100 µM Al for 24 h and 48 h. Based on the results of this prelim experiment, 100 µM Al was chosen for further experiments. Similarly, concentrations were chosen for Mn. For Al–Mn interaction study, 6-d-old seedlings were grown in Hoagland nutrient solution harbouring 500 or 1000 µM Mn in the presence or absence of 100 µM Al for 48 h, then rice plant growth, root length, shoot length, hydrogen peroxide were scored. Al and Mn were supplied in the form of AlCl_3_ and MnCl_2_, respectively. The initial pH for each solution was adjusted to 4.5.

#### Impact of Al-GSH interaction on growth physiology

For all Al–GSH interaction studies, ten seedlings were grown in nutrient solution. After CaCl_2_ treatment as described in previous section, in order to evaluate the role of GSH in attenuating Al toxicity, GSH (1 mg/L) was added at the start of Al treatment. Based on experiments performed, as described in previous section, 100 µM dose for Al stress whereas Mn dose of 1 mg/L was chosen after running a test on a range of concentrations. For the Al–Mn interaction studies, 6-d-old seedlings were grown in 0.5 mM CaCl2 solution containing 1 mg/L Mn in the presence or absence of 100 µM Al for 48 h, then rice growth, root length and H_2_O_2_ content were scored after 48 h.

### Statistical analysis

Each experiment was repeated thrice and data presented are mean ± standard error (SE). The results went through one-way ANOVA. Tukey test was performed for comparison among the set of experiments. The data analysis was carried out using Microsoft Office Excel 2013, statistical software SPSS 20 and Origin Lab8.5.

## Supplementary information

Supplementary
