# Supplementary material for: Redox balance, metabolic fingerprint and physiological characterization in contrasting North East Indian rice for Aluminum stress tolerance
Source: Sci Rep. 2019 Jun 18;9:8681. doi: 10.1038/s41598-019-45158-3 (PMC6581886; doi:10.1038/s41598-019-45158-3)
Supplement: Supplementary file 1 — Supplementary [file 41598_2019_45158_MOESM1_ESM.docx]

**Redox balance, metabolic fingerprint and physiological characterization in contrasting North East Indian rice for Aluminum stress tolerance**

**Jay Prakash Awasthi^1^, Bedabrata Saha^1^, Jogeswar Panigrahi^2^, Emiko Yanase^3^, Hiroyuki Koyama^3^, Sanjib Kumar Panda^1, *^**

^1^Assam University, Department of Life Science and Bioinformatics, Plant Molecular Biotechnology Lab, Silchar, 788011, India

^2^Khallikote University, Department of Bioscience and Bioinformatics, Berhampur, 760001, India

^3^Gifu University, Faculty of Applied Biological Sciences, Gifu, 5011193, Japan

^*^drskpanda@gmail.com

**Figure S1.**

Effect of Al dose on Shoot length (**a**) root: shoot ratio (**b**) of rice seedlings. The rice seedlings exposed to 0, 25, 50, 100 µM Al with 0.5mM CaCl_2_ pH 4.5 for 24 and 48 h. Data represent the mean root length value ± SE of ten seedlings. Differences between control and stress plants were significant at *P* < 0.05 (*) by Tukey test.





**Figure S2.**

Effect of Al stress, on the chlorophyll ‘a’ content (**a**) chlorophyll ‘b’ content (**b**) and carotenoids (**c**) in the shoot of the two rice varieties. Seedlings were exposed to AlCl_3_, containing 0.5 mM CaCl_2_ (pH 4.5) for 48 h. Values are mean ± SE (n = 3) of three separate experiments. Means denoted by the same letter were not significantly different at P< 0.05 according to Tukey multiple range test.





**Figure S3.**

Effects of Al on the content of protein carbonyl and LOX in root (**a, c**) and shoot (**b, d**) of the two rice varieties. Seedlings were exposed to 0.5 mM CaCl_2_ (pH 4.5) containing 0, 25, 50,100 µM AlCl_3_ for 48 h. Values are mean ± SE (n = 3) of three separate experiments. Means denoted by the same letter were not significantly different at P<0.05 according to Tukey multiple range test.

**

**

**Figure S4.**

Effect of Al, on the callose content for 48hr (**a**) lignin content (**b)** in the two rice varieties. Seedlings were exposed to 0,100 µM AlCl_3_, containing 0.5 mM CaCl_2_ (pH 4.5) for 48 h. Values are mean ± SE (n = 3) of three separate experiments. Means denoted by the same letter were not significantly different at P< 0.05 according to Tukey multiple range test.

**

**

**Figure S5**

Effect of Al on plant growth of rice cultivars (Al tolerant on left; Al sensitive on right the seedling were grown for 45 day in a pot with normal soil contained 0 ,100 µM AlCl_3_ in 0.5mM CaCl_2_ at pH4.5. Growth pattern of Al-tolerant Rice cultivar, Disang, (a), and Al-sensitive cultivar, Joymati (b).

**
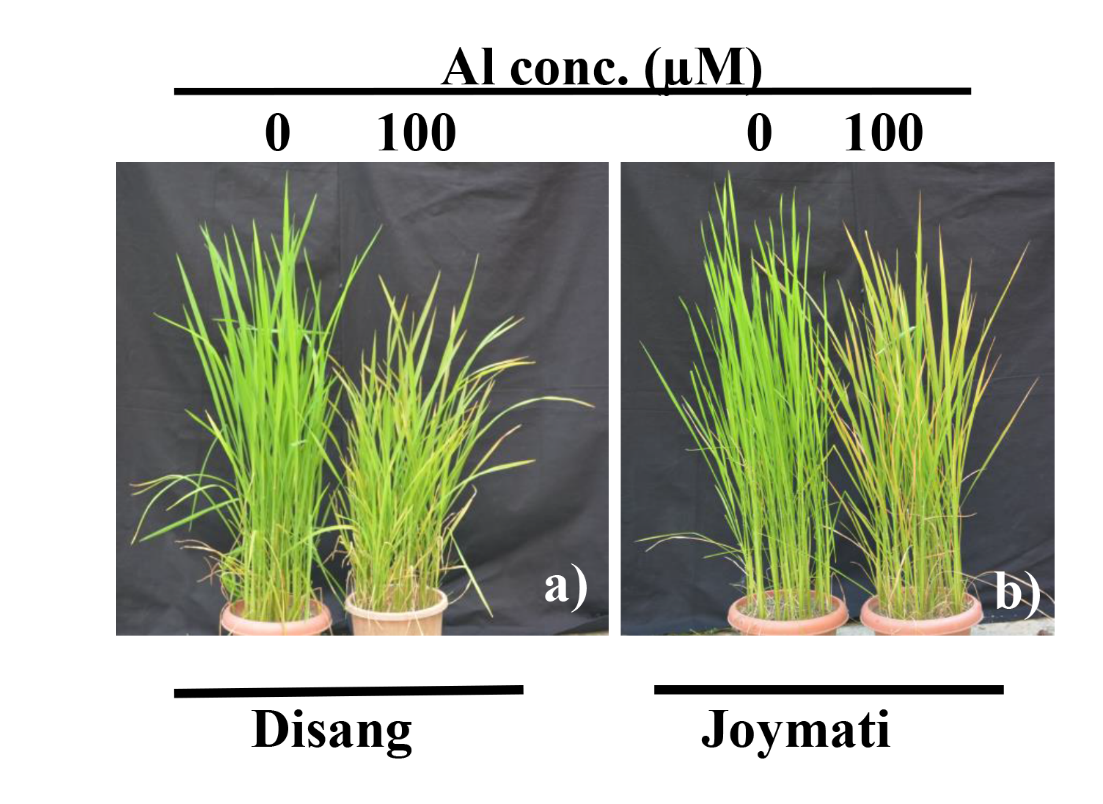
**

**Figure S6**

Effect of Al, on the protein content in root (a) and shoot (b) for 48hr in the two rice varieties. Seedlings were exposed to 0,100 µM AlCl3, containing 0.5 mM CaCl_2_ (pH 4.5) for 48 h. Values are mean ± SE (n = 3) of three separate experiments. Means denoted by the same letter were not significantly different at P< 0.05 according to Tukey multiple range test.

**

**

**Figure S7**

Al toxicity, and it’s repercussions on the protein profile of rice shoot samples. Seedlings were exposed to 0, 25, 50, 100 µM AlCl_3_, containing 0.5 mM CaCl_2_ (pH 4.5) for 48 h.

**
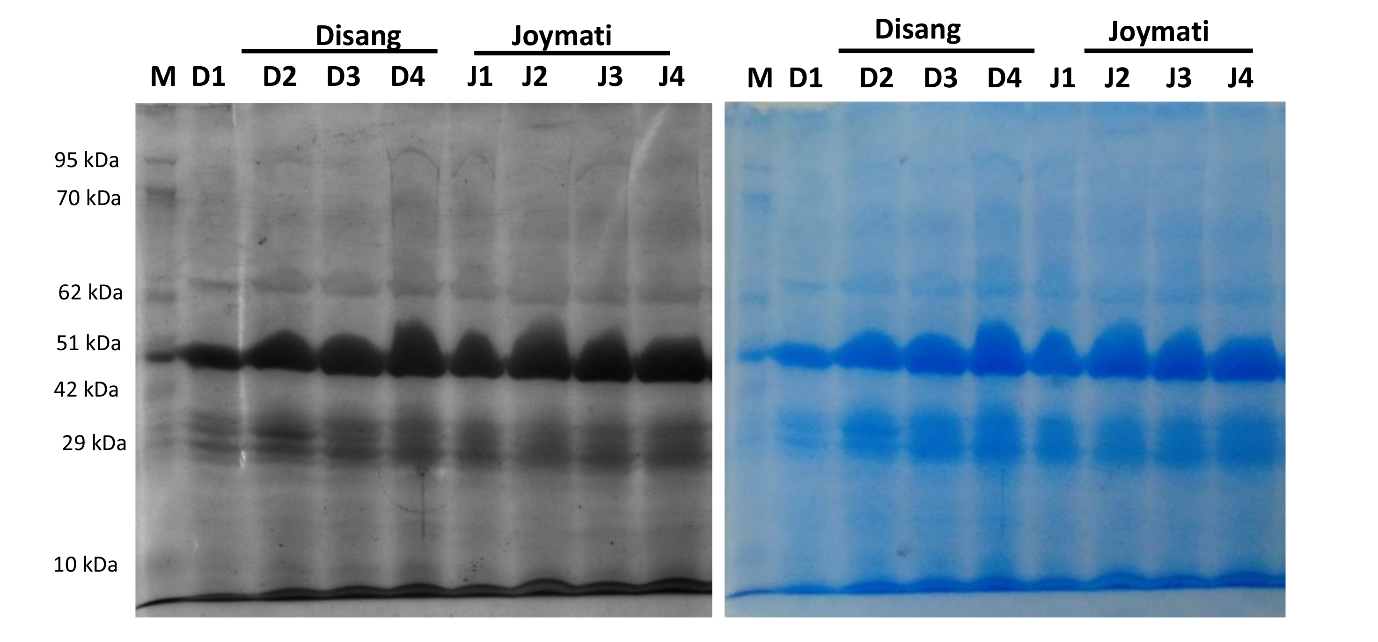
**

**Figure S8**

Malate exudation as affected by the Al (0 and 100 µM AlCl_3_) treatment in rice genotypes at 24h and 48h. Seedlings were exposed to 0, 100 µM AlCl_3_, containing 0.5 mM CaCl_2_ (pH 4.5) for 48 h. Values are mean ± SE (n = 3) of three separate experiments. Means denoted by the same letter were not significantly different at P< 0.05 according to Tukey multiple range test.

**
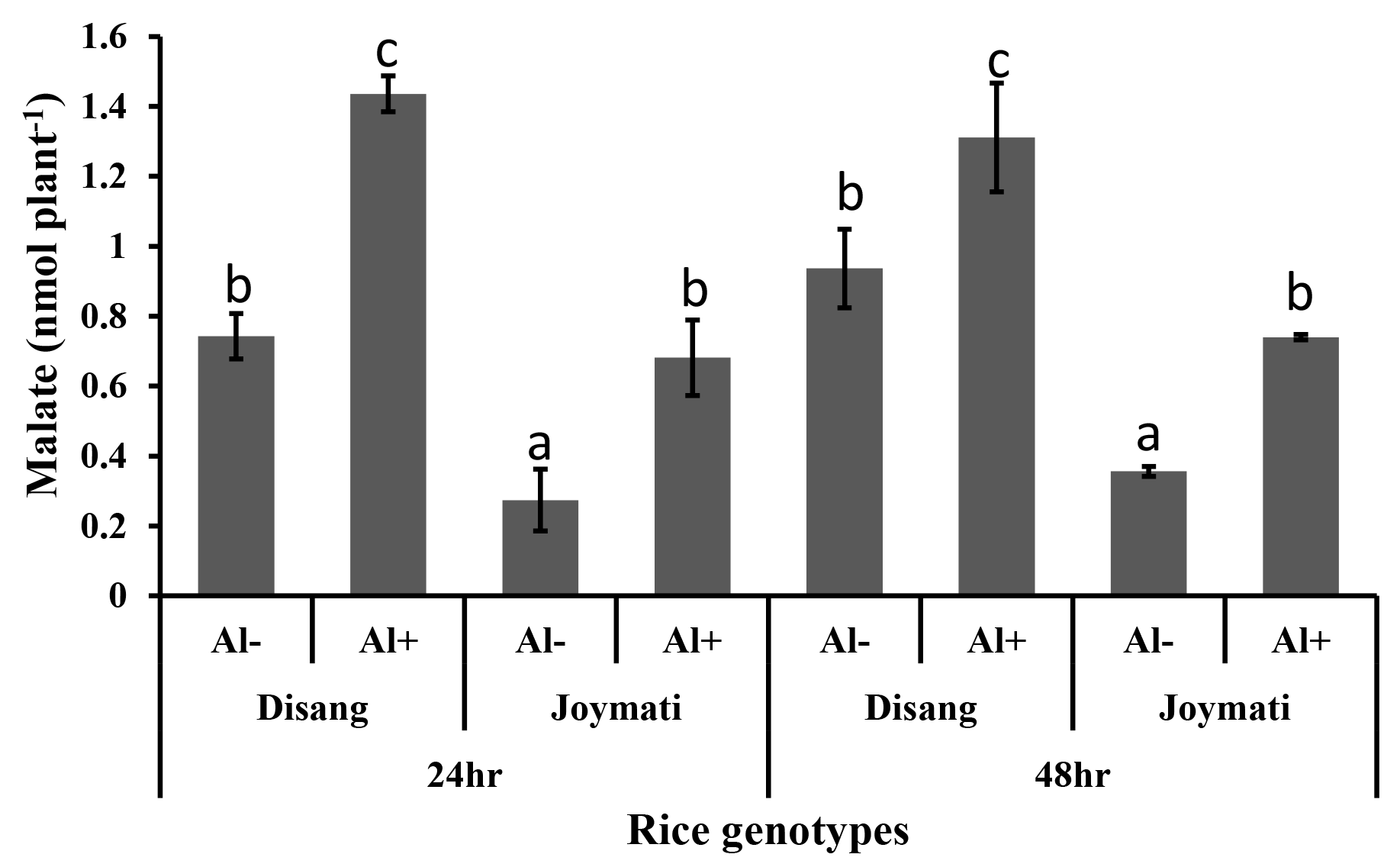
**

**Figure S9**

Native PAGE, stained for different isozyme such as SOD, APX, POX, GR, CAT isoenzyme activities in rice **root** and **shoot** under aluminum stress containing 500 µM CaCl_2_**. Lane 1,2,3,4-** Disang (0,25,50,100 µM Al Cl_3_ with CaCl_2_, **Lane 5,6,7,8** Joymati (0,25,50,100 µM AlCl_3_ with CaCl_2_) at 48hr interval.

**
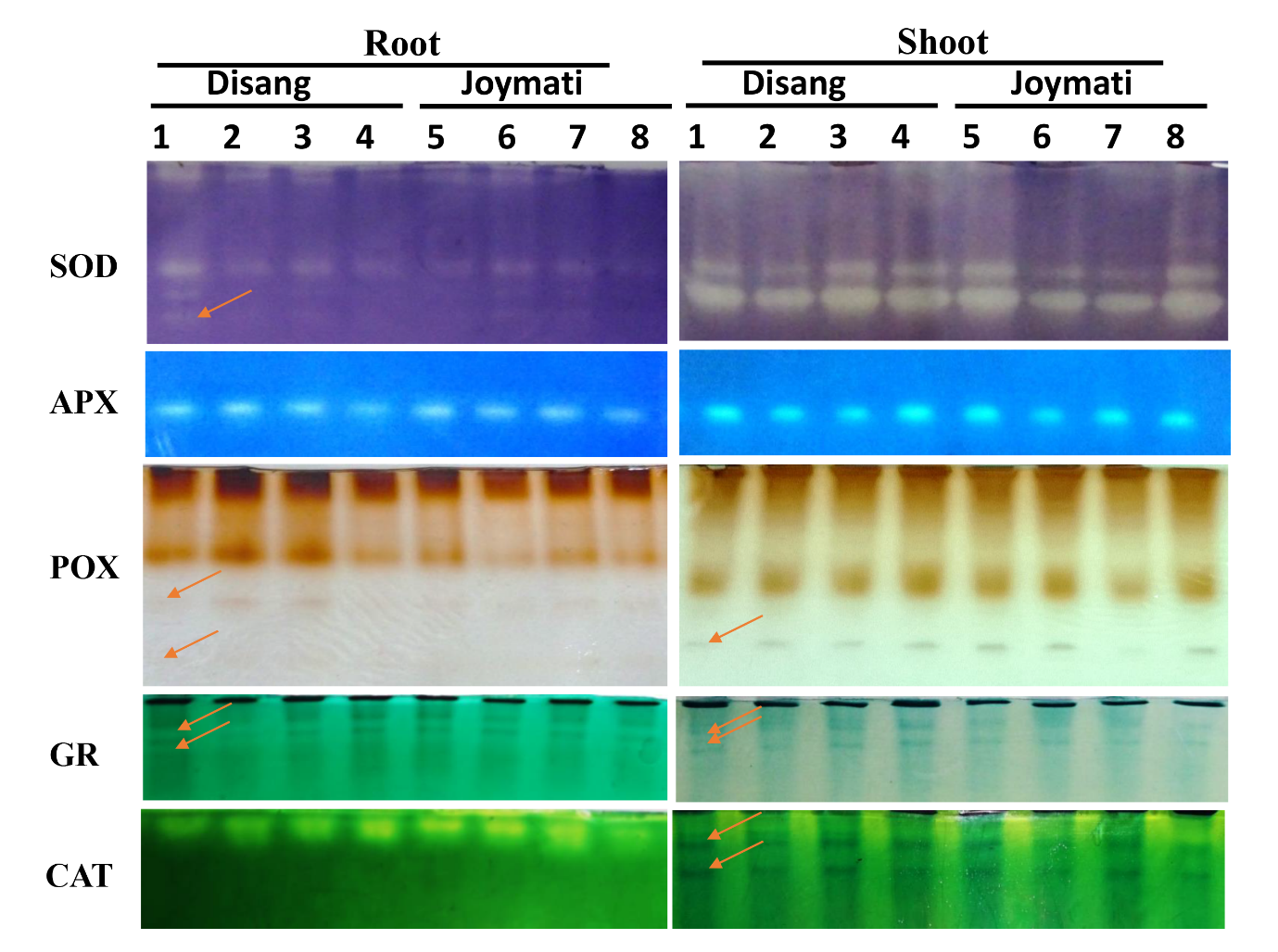
**

**Figure S10**

Effect of Al on rice seedling under Mn toxicity in a long-term experiment. Toxicity symptoms of Mn toxicity and ameliorating effect of Al on rice root and shoot length. Rice seedlings (6 day old) were grown in nutrient solution with 18, 500, 1000 µM Mn in the presence or absence of 100µM Al for 5 day (a, b). Effects of Al-GSH on rice root and shoot under Al toxicity. GSH ameliorate the effect of Al on rice root and shoot length. Rice seedlings (6 days old) were grown in nutrient solution with 0,100 µM Al and 1 mM GSH in the presence or absence of GSH for 48h (c, d). Values are mean ± SE (n = 3) of three separate experiments. Means denoted by the same letter were not significantly different at P< 0.05 according to Tukey multiple range test.

**

**

**Figure S11**

Effect of the Al-Mn interaction and Al-GSH interaction study on H_2_O_2_ content measurement of rice shoot sample (a,b) under Al toxicity. Values are mean ± SE (n = 3) of three separate experiments. Means denoted by the same letter were not significantly different at P< 0.05 according to Tukey multiple range test.

**

**

**Table S1.** : Chlorophyll fluorescence studies in both rice varieties under Al treatment for 5 days. For control populations and treated populations, *n* = 6 for each genotypes. Data shown are representative of two separate experiments. (*) represent Correlation is significant (p<0.05) level.

| *Parameter* | *Fo* | *Fm* | *Fv/Fm* | *Fm'* | *Y (II)* | *ETR* | *Fo'* | *Fv'/Fm'* | *qP* | *qN* | *qL* | *NPQ* | *Y (NO)* | *Y (NPQ)* |
| --- | --- | --- | --- | --- | --- | --- | --- | --- | --- | --- | --- | --- | --- | --- |
| Fo | 1 |  |  |  |  |  |  |  |  |  |  |  |  |  |
| Fm | -0.864 | 1 |  |  |  |  |  |  |  |  |  |  |  |  |
| Fv/Fm | -0.929* | 0.988* | 1 |  |  |  |  |  |  |  |  |  |  |  |
| Fm' | -0.738 | 0.771 | 0.766 | 1 |  |  |  |  |  |  |  |  |  |  |
| Y (II) | -0.589 | 0.801 | 0.751 | 0.931* | 1 |  |  |  |  |  |  |  |  |  |
| ETR | -0.312 | 0.532 | 0.460 | 0.861 | 0.929* | 1 |  |  |  |  |  |  |  |  |
| Fo' | 0.830 | -0.750 | -0.810 | -0.286 | -0.223 | 0.147 | 1 |  |  |  |  |  |  |  |
| Fv'/Fm' | -0.975* | 0.879 | 0.939* | 0.608 | 0.503 | 0.176 | -0.930* | 1 |  |  |  |  |  |  |
| qP | -0.313 | 0.534 | 0.462 | 0.861 | 0.929* | 0.999* | 0.145 | 0.177 | 1 |  |  |  |  |  |
| qN | 0.664 | -0.252 | -0.392 | -0.078 | 0.203 | 0.436 | 0.726 | -0.681 | 0.435 | 1 |  |  |  |  |
| qL | -0.051 | 0.354 | 0.252 | 0.688 | 0.826 | 0.961* | 0.353 | -0.069 | 0.961* | 0.666 | 1 |  |  |  |
| NPQ | 0.882 | -0.687 | -0.777 | -0.334 | -0.186 | 0.157 | 0.970* | -0.942* | 0.156 | 0.854 | 0.398 | 1 |  |  |
| Y (NO) | -0.125 | -0.245 | -0.123 | -0.529 | -0.728 | -0.884 | -0.447 | 0.217 | -0.884 | -0.804 | -0.977* | -0.527 | 1 |  |
| Y (NPQ) | -0.118 | 0.312 | 0.238 | 0.756 | 0.811 | 0.970* | 0.374 | -0.043 | 0.969* | 0.541 | 0.972* | 0.362 | -0.911* | 1 |

**Table S2:** LCMS (MS type-MS^2^) analysis of rice varieties under Al stress

| **MS2 (m/z)** | **Compound name** | **Structure** | **Molar Mass (g·mol^−1^)** | **Function** |
| --- | --- | --- | --- | --- |
| 195 | D-Gluconic acid | **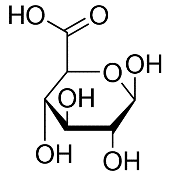** | 196.16 | It is an organic compound which are responsible for the making the chelate compound with Al^3+^ |
| 243 | Uridine | **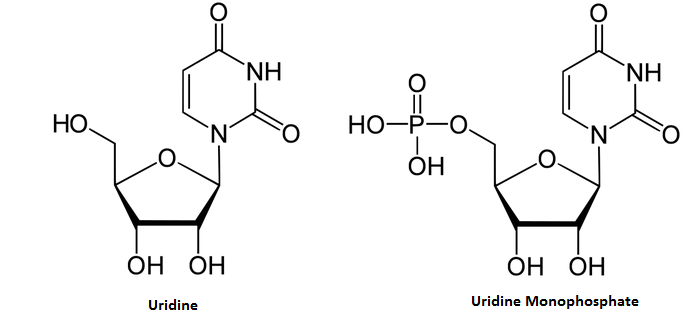** | 244.20 | Uridine is a glycosylated pyrimidine-analog containing uracil attached to a ribose ring (or more specifically, a ribofuranose) via a β-N1-glycosidic bond. |
| 275 | L- Sacchropine | **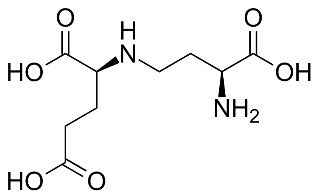** | 276.29 | It is an intermediate in the metabolism of amino acid lysine. It is a precursor of lysine in the alpha-aminoadipate pathway. In plants it is an intermediate in the degradation of lysine, formed by condensation of lysine and alpha-ketoglutarate. |
| 290 | Catechin | **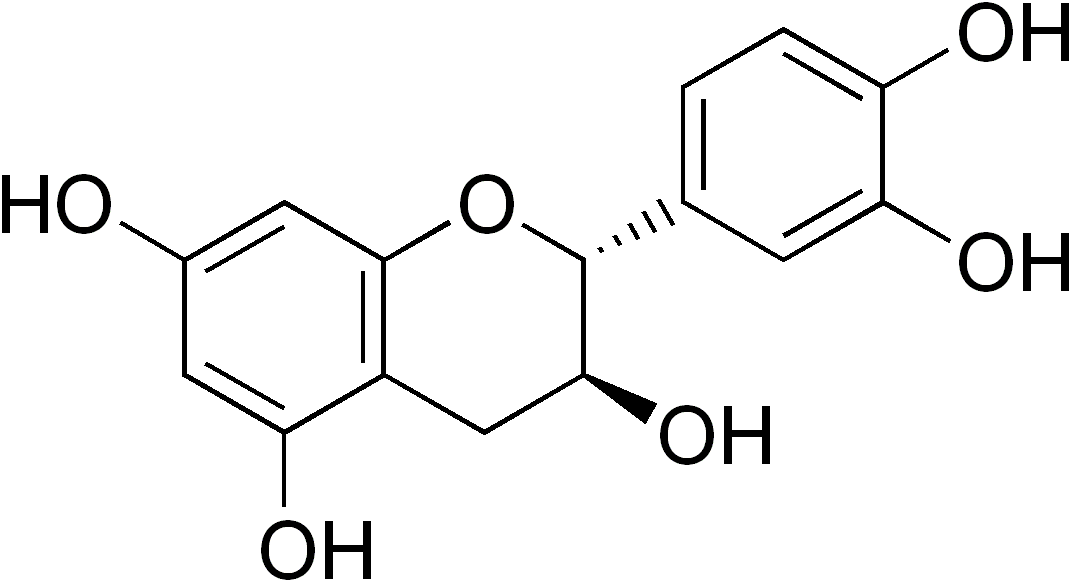** | 290.27 | It is a type of natural phenol and antioxidant. The ability to quench singlet oxygen seems to be in relation with the chemical structure of catechin. It is also found as glycoside and the glycosidic form has also antioxidant properties. |
| 540 | Unknown | ? | ? | ? |

Use Mass bank data base: A public repository for sharing mass spectral data for life sciences. (Http://www.massbank.jp/SearchPage.html)
